# Excited state non-adiabatic dynamics of large photoswitchable molecules using a chemically transferable machine learning potential

**DOI:** 10.1038/s41467-022-30999-w

**Published:** 2022-06-15

**Authors:** Simon Axelrod, Eugene Shakhnovich, Rafael Gómez-Bombarelli

**Affiliations:** 1 Department of Chemistry and Chemical Biology, Harvard University, Cambridge, MA 02138 USA; 2 Department of Materials Science and Engineering, Massachusetts Institute of Technology, Cambridge, MA 02139 USA

**Keywords:** Molecular dynamics, Excited states, Chemical physics, Computational chemistry

## Abstract

Light-induced chemical processes are ubiquitous in nature and have widespread technological applications. For example, photoisomerization can allow a drug with a photo-switchable scaffold such as azobenzene to be activated with light. In principle, photoswitches with desired photophysical properties like high isomerization quantum yields can be identified through virtual screening with reactive simulations. In practice, these simulations are rarely used for screening, since they require hundreds of trajectories and expensive quantum chemical methods to account for non-adiabatic excited state effects. Here we introduce a *diabatic artificial neural network* (DANN), based on diabatic states, to accelerate such simulations for azobenzene derivatives. The network is six orders of magnitude faster than the quantum chemistry method used for training. DANN is transferable to azobenzene molecules outside the training set, predicting quantum yields for unseen species that are correlated with experiment. We use the model to virtually screen 3100 hypothetical molecules, and identify novel species with high predicted quantum yields. The model predictions are confirmed using high-accuracy non-adiabatic dynamics. Our results pave the way for fast and accurate virtual screening of photoactive compounds.

## Abstract (web summary)

The authors introduce a diabatic neural network to accelerate excitedstate, non-adiabatic simulations of azobenzene derivatives. The model predicts quantum yields for unseen species that are correlated with experiment.

## Introduction

Light is a powerful tool for manipulating molecular systems. It can be controlled with high spatial, spectral and temporal precision to facilitate a variety of processes, including energy transfer, intermolecular reactions, and photoisomerization^1^. These processes are used in areas as diverse as synthesis, energy storage, display technology, biological imaging, diagnostics and medicine^1–3^. Photoactive drugs, for instance, are photoswitchable compounds whose bioactivity can be toggled through light-induced isomerization. Precise spatiotemporal control of bioactivity allows photoactive drugs to be delivered in high doses with minimal off-target activity and side effects. Such therapeutics are a promising path for the treatment of cancer, neurodegenerative diseases, bacterial infections, diabetes, and blindness^4,5^.

Theory plays a key role in explaining and predicting photochemistry because empirical heuristics learned from thermally activated ground state processes typically do not apply to excited states^3^. Computer simulations based on quantum mechanics can achieve impressive accuracy in the prediction of experimental observables. These include the isomerization efficiency and absorption spectrum of photoswitchable compounds^6^, which are key quantities in the design of photoactive drugs.

However, ab initio methods in photochemistry are severely limited by their computational cost^7^. In order to gather meaningful statistics for one molecule, hundreds of replicate simulations are needed, each of which involves thousands of electronic structure calculations performed in series with sub-femtosecond timesteps. The individual quantum chemical calculations are particularly demanding, requiring excited state gradients and some treatment of multireference effects. In some cases, both the ground and excited state gradients are required at each time step^8,9^. Using ab initio methods to compute photochemical properties of tens or hundreds molecules is impractical, and photodynamic simulations have not yet been used for large-scale virtual screening.

Among the most accurate and expensive electronic structure methods are multireference perturbation techniques^10–15^, but their cost and requirement for manual active space selection limit their use in virtual screening. The photochemistry community has made exciting developments over several years to overcome both of these hurdles. For example, reduced scaling techniques^16,17^ and graphics processing units^18^ can significantly accelerate multi-reference calculations. The density matrix renormalization group (DMRG)^19,20^ and multi-reference density functional theory (DFT) methods^21–23^ have expanded the size of systems that can be treated with high accuracy. DMRG has also been used to automate the selection of active spaces for multi-reference methods^24,25^. Less accurate but more affordable black-box methods include spin-flip time-dependent DFT (SF-TDDFT)^26^ and hole-hole Tamm-Dancoff DFT^27^, among others^28–33^. Despite these developments, the cost of non-adiabatic simulations remains high. As discussed below, even SF-TDDFT is prohibitively expensive for virtual screening. Semi-empirical methods^34–36^ are currently the only affordable approach for large-scale screening. They provide qualitatively correct results across many systems, but are ultimately bounded by their approximations, with average energy errors of 15 kcal/mol^35^.

A different approach is to use data-driven models in place of quantum chemistry (QC) calculations. Machine learning (ML) models trained on quantum chemical data can now routinely predict ground state energies and forces with sub-chemical accuracy^37–39^, and take only milliseconds to make predictions. These models have been successfully used in a variety of ground state simulations^38,40,41^. They have also been used to accelerate non-adiabatic simulations in a number of model systems^42–48^. However, excited state ML has not yet offered affordable photodynamics for hundreds of molecules of realistic size, which is the ultimate goal for predictive simulation in photopharmacology. Further, no excited state interatomic potentials have been developed that are transferable to different compounds. They therefore require thousands of QC calculations for every new species to serve as training data.

Here we make significant progress toward affordable, large-scale photochemical simulations and virtual screening with ML. To develop a transferable potential we focus on molecules from the same chemical family, studying derivatives of azobenzene, a prototypical photoswitch. The derivatives studied here contain up to 100 atoms, making them the largest systems fit with excited state ML potentials to date. Combining an equivariant neural network^38^ and a physics-informed diabatic model, together with data generated by combinatorial exploration of chemical space, and configurational sampling through active learning, we produce a model that is transferable to large, unseen derivatives of azobenzene. This yields computational savings in excess of six orders of magnitude. Predicted isomerization quantum yields of unseen species are correlated with experimental values. The model is used to predict the quantum yield for over 3100 hypothetical species, revealing rare molecules with high *cis*-to-*trans* and *trans*-to-*cis* quantum yields.

## Results

### Azobenzene photoswitches

**Fig. 1:**
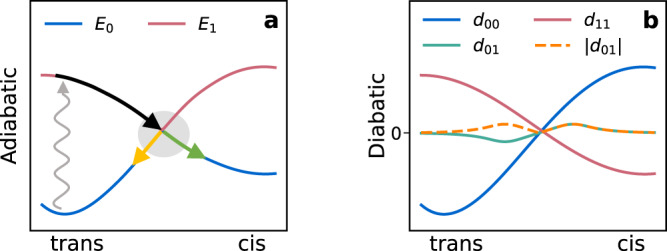
Depiction of the potential energy surfaces in azobenzene derivatives. **a** *S*_0_ and *S*_1_ adiabatic energies, with the CI region shaded in gray. Initial excitation is shown with a vertical zigzag line. Trajectories prior to hopping are shown in black. Reactive and unreactive trajectories after hopping are shown in green and yellow, respectively. **b** Diabatic energies ${d}_{nm}\equiv {({{{\mathbf{H}}}}_{d})}_{nm}$. The diagonal diabatic elements cross and become re-ordered along the isomerization coordinate. A CI occurs when the diagonal diabatic elements cross and the off-diagonal element becomes zero.

**Fig. 2:**
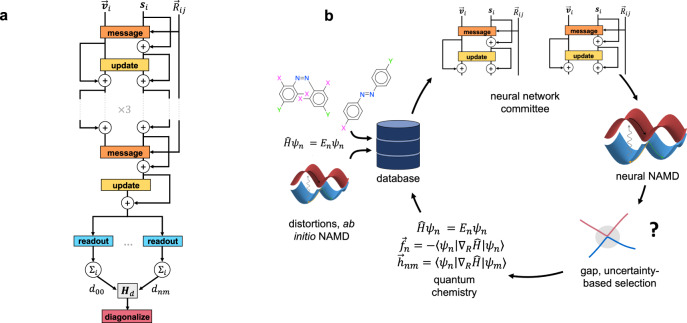
Neural network architecture and active learning loop. **a** Schematic of the DANN architecture, which is based on the PaiNN model. Scalar atomic features **s**_*i*_ and vectorial atomic features ${\overrightarrow{{{{{{{{\bf{v}}}}}}}}}}_{i}$ are updated through messages from neighboring atoms. The **s**_*i*_ are then mapped to atomic energies, which are summed to produce the diabatic Hamiltonian ${{{\bf{H}}}}_{d}$. The diabatic matrix is diagonalized to produce adiabatic quantities. **b** Schematic of the active learning loop. Geometries and QC data are first generated through ab initio NAMD, normal mode sampling, and inversion/rotation about the central N=N double bond. Two neural networks are then trained on the data and used to perform DANN-NAMD. Newly generated geometries with high committee variance and/or low predicted gaps receive QC calculations. The new calculations are added to the training data, the networks are retrained, and the cycle is repeated until convergence.

This work focuses on the photoswitching of azobenzene derivatives, but the methods are general and can be applied to other chemistries and other excited state processes. Azobenzene derivatives can exist as *cis* or *trans* isomers. The conformations are local minima in the ground state, but not in the excited state. Photoexcitation of either can therefore induce isomerization into the other (see the potential energy schematics in Figs. 1(a) and 2(b)). A key experimental observable is the quantum yield, defined as the probability that excitation leads to isomerization. The yield depends critically on the dynamics near conical intersections (CIs), configurations in which the excitation energy is zero. In these regions the electrons can return to the ground state with non-zero probability.

Many approaches have been developed over several decades to model such non-adiabatic transitions. These include ab initio multiple spawning^49^ and cloning^50^; Ehrenfest dynamics^8,9^; coherent switching with decay of mixing^51^; the variantional multi-configurational Gaussian method^52^; exact factorization^53–57^; the multi-configuration time-dependent Hartree (MCTDH) method^58,59^; Gaussian MCTDH^60^; and trajectory surface hopping^61^. A recent review of these methods can be found in ref. ^3^. Surface hopping is a popular approach because of its simplicity and efficiency. In this method, independent trajectories are simulated with stochastic hops between potential energy surfaces (PESs). Depending on the curvature of the PESs and the location of the hop, a trajectory can end in the original isomer or in a new isomer (Figs. 1(a) and 2(b)). The quantum yield is the proportion of trajectories that end in a new isomer. Our goal is to predict the quantum yield of azobenzene derivatives after excitation from the singlet ground state (*S*_0_) to the first singlet excited state (*S*_1_). This can be accomplished with the surface hopping approach described above, using a fast surrogate ML model to generate the PESs. The impact of considering only the first excited state is discussed in Supplementary Note 3.

### ML architecture and training

Our model is based on the PaiNN neural network^38^, which uses equivariant message-passing to predict molecular properties. In this approach, an initial feature vector is generated for each atom using its atomic number. The vector is then updated through a set of neural network operations involving “messages”, which incorporate the distance, orientation, and features of atoms within a cutoff distance. A series of updates leads to information being aggregated from increasingly distant atoms. Once the updates are complete, the atomic features are mapped to molecular energies using a neural network.

This architecture can be used to predict energies and, through automatic differentiation, the forces for each state. However, models that predict adiabatic energies have a basic shortcoming for non-adiabatic molecular dynamics (NAMD). Since surface hopping is largely controlled by the energy gap when it is close to zero, small errors in the energies can lead to exponentially large errors in the hopping probability^62,63^. This in turn can cause large errors in observable quantities like the quantum yield. This point is discussed in further detail in Supplementary Methods A. Further, since CIs are non-differentiable cusps in the energy gap, they are difficult to fit with neural networks. For *N* atoms in a molecule, the network must predict two different energies that are exactly equal in 3*N* − 8 dimensions. We found this to be particularly challenging for *trans* species that are outside the training set. As shown in Supplementary Note 6, small errors in the gap lead to the incorrect prediction that many species never hop to the ground state.

To remedy this issue we introduce a model based on diabatic states, which we call DANN (diabatic artificial neural network; Fig. 2(a)). The approach builds on previous work using neural networks for diabatization^64–66^. Much of the previous work could only be used for specific system types, such as semi-rigid molecules^65^ and coupled monomers, and is thus not applicable to azobenzene. None of the methods have been used for large systems with significant conformational changes^64,66^, such as azobenzene derivatives. Further, our work uses diabatization to ease the fitting of adiabatic states across chemical space. In particular, it addresses the issue of gap overestimation near conical intersections of unseen species, as described in Supplementary Notes 1 and 6. Our work uses diabatization to address this problem, whereas previous work only used diabatization in single, model species. We also note that gap overestimation in unseen species is both a newly-identified and newly-addressed problem, as previous work in ML-NAMD focused on single species only^42–48^.

The diabatic energies form a non-diagonal Hamiltonian matrix, ${{{\mathbf{H}}}}_{d}$, which is diagonalized to yield adiabatic energies. When a 2 × 2 sub-block of ${{{\mathbf{H}}}}_{d}$ has diagonal elements that cross, and off-diagonal elements that pass through zero, a CI cusp is generated (Fig. 1). The diabatic energies that generate the cusp are smooth, which makes them easier to fit with an interpolating function than the adiabatic energies. In the DANN architecture, smoothness is imposed through a loss function related to the non-adiabatic coupling vector (NACV). The loss minimizes the value that the NACV takes when it is rotated from the adiabatic basis (Eq. (3)) into the diabatic basis. The NACV measures the change in overlap between two wavefunctions after a small nuclear displacement. If the NACV between two states is zero, then their wavefunctions must change slowly in response to a nuclear perturbation. Therefore, their energies cannot form the cusp in Fig. 1(a), and must instead resemble the smooth energies in Fig. 1(b).

The DANN model was trained on SF-TDDFT^26^ calculations for 567,037 geometries, using the 6-31G* basis^67^ and BHHLYP^68^ exchange-correlation functional. Unlike traditional TDDFT^69^, SF-TDDFT provides an accurate description of the CI region^70^, and, unlike multi-reference methods, is fairly fast and requires no manual parameter selection. The configurations were sampled from 8,269 azobenzene derivatives, of which 164 were taken from the experimental literature. The remaining molecules were generated from combinatorial substitution using common literature patterns (Supplementary Tables X and XI).

The data generation process is shown in Fig. 2. Initial data was generated through ab initio NAMD with 164 species from the literature, together with normal-mode sampling and distortions of the combinatorial species to near-CI regions. The remaining data was generated through active learning. In each cycle we trained a committee of models, used one model to perform NN-NAMD, and used the committee variance and energy gap to choose NAMD geometries for new quantum chemistry calculations. The cycle was repeated five times in total; further details can be found in the Methods section.

### Validation

To test whether the model could reproduce experimental results for unseen molecules, we evaluated it on species that were outside the training set. The test set contained 40 species (20 *cis*/*trans* pairs), including 33 with experimental *S*_1_ quantum yields in non-polar solution. Non-polar solution was chosen because it is the closest to the gas-phase conditions simulated here. Solvent effects can be easily incorporated into the model through transfer learning to implicit solvent calculations. This was previously shown to require new calculations for only a small proportion of the training set^40^.

The performance of the model is summarized in Table 1. Statistics are shown for both seen and unseen species. The former contains species that are in the training set, but geometries that are outside of it. The geometries were selected with the balanced sampling criteria described in Supplementary Note 9. Geometries from unseen species were generated with DANN-NAMD using the final trained model. Half of the DANN-NAMD geometries were selected randomly from the full trajectory and half by proximity to a CI (Supplementary Eq. (13)). 100 configurations were chosen for each molecule.

**Table 1:** MAE and coefficient of determination (*R*^2^) of the DANN model for various quantities.

		*E*_0_	*E*_1_	Δ*E*_01_	${({{\Delta }}{E}_{01})}_{{{{{{{{\rm{small}}}}}}}}}$^*a*^	${\overrightarrow{f}}_{0}$	${\overrightarrow{f}}_{1}$	${\overrightarrow{h}}_{01}$
Seen species	MAE (*↓*)	0.86	1.01	0.75	0.47	1.00	1.17	0.87
	*R*^2^ (*↑*)	1.00	1.00	1.00	0.97	0.99	0.99	0.84
Unseen species	MAE (*↓*)	3.06	3.77	1.89	0.97	1.72	2.31	1.36
	*R*^2^ (*↑*)	0.99	0.98	0.98	0.95	0.97	0.86	0.50

^*a*^For these *R*^2^ calculations, we computed the total sum of squares using mean{Δ*E*_01_} instead of ${{{{{{{\rm{mean}}}}}}}}\{{({{\Delta }}{E}_{01})}_{{{{{{{{\rm{small}}}}}}}}}\}$. The mean predictor should not know a priori which gaps are small, and hence should predict the mean of all gaps.

Units are kcal/mol for energies and kcal/mol/Å for forces and force couplings. *E*_*i*_ are energies, ${\overrightarrow{f}}_{i}$ are forces, Δ*E*_01_ is the energy gap, and ${\overrightarrow{h}}_{01}$ is the force NACV. ${({{\Delta }}{E}_{01})}_{{{{{{{{\rm{small}}}}}}}}}$ denotes the energy gap when it is under 4.6 kcal/mol (0.2 eV).

For species in the training set, all quantities are accurate to within approximately 1 kcal/mol(/Å). Apart from the NACV, all quantities have *R*^2^ correlation coefficients close to 1. The *R*^2^ of the NACV is 0.84. This may be somewhat low because diabatization cannot remove the curl component of the NACV in the diabatic basis^71,72^. This would also explain the low *R*^2^ value for the NACV in ref. ^45^, which computed it as the gradient of a scalar. For molecules outside the training set, all quantities apart from the energies have an error below 3 kcal/mol(/Å). The energy gaps and ground state forces have *R*^2^ correlation coefficients near 1. The gap error of 1.89 kcal/mol should be contrasted with the error of 15 kcal/mol for the semi-empirical method in ref. ^35^. The errors in the excited state forces are slightly larger, but still quite low. The correlation coefficient for the force NACV ${\overrightarrow{h}}_{01}$ is rather poor. As described in Supplementary Note 6, the yields of *trans* derivatives are better correlated with experiment when using Zhu-Nakamura surface hopping than Tully’s method. The latter uses the NACV and the former does not, so part of the difference may be explained by the high error in the force NACV. Nevertheless, there is still reasonable agreement between Tully’s method and experiment, suggesting that errors in the force NACV do not spoil the dynamics.

**Fig. 3:**
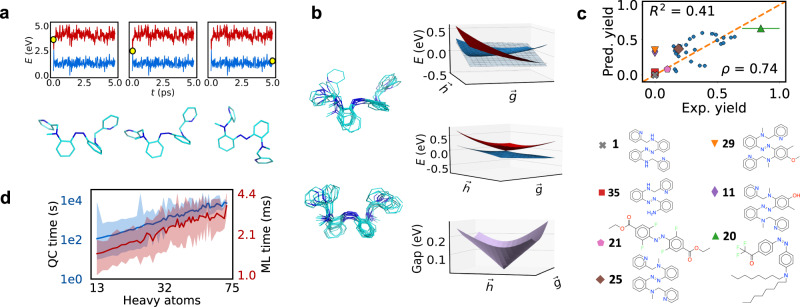
Speed and accuracy of DANN-NAMD. **a** Selected trajectory frames for a molecule outside the training set. The top panels show the *S*_0_ and *S*_1_ energy as a function of time. A yellow dot indicates the time at which the snapshot below was taken. **b** Left: Overlay of selected hopping geometries from reactive (top) and unreactive (bottom) trajectories. Right: PES as a function of branching plane coordinates at one of the reactive hopping geometries. Diabatic energies, adiabatic energies, and adiabatic gaps are shown from top to bottom. The diabatic coupling is shown in gray. **c** Predicted vs. experimental quantum yield for 33 species outside the training set. The *R*^2^ value and Spearman rank correlation *ρ* are both shown. Color-coded data points are defined below. Prediction error bars represent one standard deviation of 1000 bootstrapped samples. Experimental error bars represent reported uncertainties or standard deviations from multiple observations, when applicable. **d** Node time for QC and ML calculations.

Figure 3(a) shows snapshots from an example DANN-NAMD trajectory, and panel (b) shows random samples of the hopping geometries. Reactive hopping geometries are shown on top, and non-reactive ones are shown below. The molecule is the (aminomethyl)pyridine derivative **26**, with the species numbering given in Supplementary Data 2 and 3. The overlays show *cis*-*trans* isomerization proceeding through inversion-assisted rotation, consistent with previous work^73^. The dominant motion is rotation, with the CNNC dihedral angle increasing in magnitude from −10^∘^ at equilibrium to −86^∘^ at the hopping points. Significant changes also occur in the CNN and NNC angles, with each transitioning from 123^∘^ to either 113^∘^ or 135^∘^.

The predicted PES in the branching space $(\overrightarrow{g},\overrightarrow{h})$ is shown beside the geometries. $\overrightarrow{h}$ is the direction of the force NACV and $\overrightarrow{g}\propto {\nabla }_{R}({{\Delta }}{E}_{01})$ is the direction of the gap gradient. Each vector was computed with automatic differentiation using Eq. (1). The diabatic energies, adiabatic energies, and gap are shown from top to bottom. We see that the model generates a true CI, in which the *S*_0_ and *S*_1_ energies are exactly equal. Further, the degeneracy is lifted in both the $\overrightarrow{g}$- and $\overrightarrow{h}$-directions, so that the *S*_1_ energy and gap each form a characteristic cone. These hallmarks of CIs are built into the model because the adiabatic energies are eigenvalues of a diabatic matrix. For example, the cone emerges from the fact that *d*_11_ − *d*_00_ and *d*_01_ each pass linearly through zero in different directions^74^.

Figure 3(c) indicates that the predicted and experimental quantum yields of unseen species are correlated. The yields are for the 33 *cis* and *trans* species with experimental data in Supplementary Data 1. The *R*^2^ value is 0.42, and the Spearman rank correlation coefficient *ρ* is 0.74. While the *R*^2^ value is somewhat low, the Spearman rank correlation is high. The Spearman coefficient measures the accuracy with which the model ranks species by quantum yield. *ρ* only compares orderings, while *R*^2^ compares the model error to the error of a mean predictor. This means that *ρ* is a more forgiving metric, and also a more relevant metric for virtual screening. Since *cis* isomers have yields two to three times higher than *trans* isomers, the high value of *ρ* means that the model properly separates the isomers into low- and high-yield groups.

Further, as shown in Supplementary Figs. 5 and 7, the model produces meaningful rankings among *trans* species. The correlation coefficients are *ρ* = 0.32 using Tully’s method^61^ and *ρ* = 0.57 using the Zhu-Nakamura approach^75^. The model is largely able to differentiate between high- and low-yield *trans* derivatives. Several such molecules are of interest. They are color-coded in the plots, with the legend given below. A full list of predictions is given in Supplementary Data 1. We see, for example, that the (aminomethyl)pyridine derivatives **1** and **35** are both predicted to have near-zero yields. These species do not isomerize from *trans* to *cis*, because strong N-H hydrogen bonds lock the planar *trans* conformation in place^76^. Replacing the NH group in **1** with N - CH_3_ gives species **25**. This molecule isomerizes because there is no hydrogen bonding. This, too, is predicted by the model. Further, the hepta-tert-butyl derivative **17** has an experimental and predicted yield of zero. This is likely because of steric interactions among the bulky tert-butyl groups. While able to account for these two different mechanisms, the model fails to predict the subtle electronic effects in species **11** and **29**. Resonance interactions between oxygen lone pairs and the azo group modify the PES, such that there is no rotational CI^77^. There is instead a concerted inversion CI, which occurs too early along the path between *trans* and *cis* to allow for isomerization. The changes in the PES may either be too small or too specific to the substituents for the model to predict without fine tuning. Finally, derivatives with high yields are partly distinguished from those with low but non-zero yields. An example is **21**, whose experimental yield of 10% is half that of *trans*-azobenzene. The model properly identifies this molecule as having a low yield, but also mistakenly does the same for several high-yield species. The accuracy for unseen species could always be improved with transfer learning, in which the model is fine-tuned with a small number of calculations from a single molecule (discussed below). This would increase the computational cost, but would still be orders of magnitude less expensive than ab initio NAMD.

**Fig. 4:**
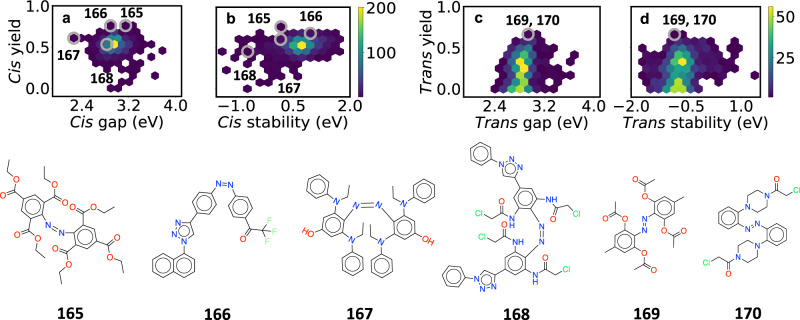
Results of virtual screening. Species of interest are circled in gray and shown below the plots. **a** Predicted yield vs. excitation energy for *cis* derivatives. **b** Predicted yield vs. stability for *cis* derivatives. **c**-**d** As in (**a**-**b**), but for *trans* derivatives.

While meaningful correlations are produced for *trans* species, the same is not true of *cis* molecules (*ρ* = 0.02). This may be because there are no *cis* derivatives with zero yield. Nevertheless, the model properly identifies **20** as having the highest yield. Further, it does not mistakenly assign a zero yield to any derivative. This is noteworthy because, as shown in Fig. 4(a) and (b), some hypothetical *cis* species are predicted to have zero yield. Synthesis of non-switching *cis* derivatives and comparison to predictions could therefore be of interest in the future.

Overall, we observe moderate correlation between predicted and experimental yields. The Spearman correlation is high when including both isomers, moderate for *trans* isomers, and low for *cis* isomers. The *R*^2^ value, a measure of numerical error compared to that of a mean predictor, is moderate when including both isomers and near-zero when separating them. Indeed, the MAEs of the mean predictor are 9.5%, 10.3%, and 17.7% for *trans*, *cis*, and all species, respectively. The model MAEs before (after) subtracting the mean signed error are 14.4% (13.5%), 11.5% (11.2%) and 13.2% (13.0%). In addition to model error, sources of error include inaccuracies in SF-TDDFT, approximations in surface hopping, solvent effects, and experimental uncertainty. These are discussed in depth in Supplementary Note 3. Each source of error affects both *R*^2^ and *ρ*, but is expected to have a larger effect on *R*^2^. The rank correlation with experiment is encouraging given the difficulty of the task, as captured by the sensitivity of the yield to model errors in the PES^75^, and given the sources of error outside the model. Further, as discussed below, DANN provides an excellent starting point for fine-tuned, molecule-specific models that can be used for high-accuracy simulations of single species.

Figure 3(d) shows that DANN-NAMD is extremely fast. The plot shows the node time, defined as *t*_calc_/*n*_calc_, where *t*_calc_ is the calculation time per geometry, and *n*_calc_ is the number of parallel calculations that can be performed on a single node. We see that ML speeds up calculations by five to six orders of magnitude. The direct comparison of the pre-trained model node times and QC node times is appropriate because the model generalizes to unseen species. This means that it incurs no extra QC cost for any future simulations. The minimum speedup corresponds to the smallest molecules (14 heavy atoms or 24 total atoms), and the maximum to the largest molecules (70 heavy atoms or 99 total atoms). This reflects the different scaling of the QC and ML calculations. Empirically we see that DANN scales as *N*^0.49^ for *N* heavy atoms, while SF-TDDFT scales as *N*^2.8^. These values come from fitting the timings to *t* = *A* ⋅ *N*^*x*^, where *t* is the computational time, *A* and *x* are fitted constants, and *N* is the number of heavy atoms. DANN’s apparent sub-linear scaling is an artifact of diagonalizing ${{{\mathbf{H}}}}_{d}$; when the diagonalization is removed, the scaling becomes linear. This is the expected scaling for a message-passing neural network with a fixed cutoff radius. Evidently diagonalizing ${{{\mathbf{H}}}}_{d}$ introduces a large overhead with weak dependence on system size. Nevertheless, we see that DANN is still quite fast.

### Virtual screening

Having shown that the model is fast and generalizes in the chemical and configurational space of azobenzenes, we next used it for virtual screening of hypothetical compounds. We first retrained the network on all available data, including species that were originally held out, for a total of 631,367 geometries in the training set. We then predicted the quantum yields of 3100 combinatorial species generated through literature-informed substitution patterns, as in ref. ^78^. This screen served two purposes. The first was to gather statistics about the distribution of photophysical properties of azobenzenes at a scale not accessible to experiments or traditional simulations. The second was to identify molecules with rare desirable properties. In particular, we sought to find molecules with high *c* → *t* or *t* → *c* quantum yields and redshifted absorption spectra. The former is important because increasing the ratio QY_*a*→*b*_/QY_*b*→*a*_, where QY is the quantum yield, can lead to more complete *a* → *b* transformation under steady state illumination. This is critical for precise spatial control of drug activity when the two isomers have different biological effects^79^. Redshifting is a crucial requirement for photo-active drugs, since human tissue is transparent only in the near-IR^79^.

The results are shown in Fig. 4. Panels (a) and (c) show the predicted yield vs. mean gap. For each species we averaged the gap over the configurations sampled during neural network ground state MD. The thermal averaging led to a typical blueshift of 0.2–0.3 eV relative to the gaps of single equilibrium geometries. The mean excitation energies are 2.95 eV for *cis* derivatives and 2.84 eV for *trans* species; the gaps are 2.98 eV and 2.97 eV for the respective unsubstituted compounds. The average gaps and their differences are similar to experimental measurements for azobenzene^80^. The average *c* → *t* and *t* → *c* yields are 54% and 24%, respectively, while those of the unsubstituted species are 59% and 37%. These are consistent with experimental results in non-polar solution, for which the base compound has yields of 44–55% and 23–28%^80^; the former is closer to 55% on average. However, the yield of the base *trans* compound is overestimated with respect to both theory and experiment^6,75,80^. The mean (median) proportion of trajectories ending in the ground state after 2 ps are 92% (100%) for *cis* species and 31% (17%) for *trans* species. The standard deviations are 25% and 30%, respectively.

Panels (b) and (d) show the yield plotted against the isomeric stability, defined as *E*_*trans*_ − *E*_*cis*_ for *trans* isomers and *E*_*cis*_ − *E*_*trans*_ for *cis* isomers. The energy *E* is the median value of the configurations sampled in the ground state; we used the median to reduce the effect of outlier geometries. On average the *trans* isomers are more stable than the *cis* isomers by 0.66 eV (15.3 kcal/mol), which is similar to experimental values over 10 kcal/mol for azobenzene^81^. The stability is of interest for three reasons. First, a large absolute value indicates that one isomer is dominant at room temperature. This is essential for photoactive drugs, and is the case for regular azobenzene. Second, an inverted stability, in which *cis* is more stable than *trans*, allows for stronger absorption at longer wavelengths. This is because the dipole-forbidden *n* − π^*^ (*S*_1_) transition is significantly stronger for *cis* than for *trans*^80^. Third, in photopharmacology, one often wants to deliver a drug in inactive form, and activate it with light in a localized region. If *trans* happens to be active and *cis* inactive, then localized activation is only possible if *cis* is more stable.

Several species of interest are shown in Fig. 4. The molecules **165** and **166** have predicted *c* → *t* yields of 75 ± 6% and 72 ± 6%, respectively, which are well above the *cis* average of 55%. The species **169** and **170** have predicted *t* → *c* yields of 66 ± 7% and 63 ± 10%, respectively, which are three times the average *trans* yield. Molecule **167** is highly redshifted, with a mean predicted gap of 2.26 eV (548 nm), and a standard deviation of 0.87 eV. QC calculations on the geometries sampled with DANN gave a gap of 2.26 ± 0.61 eV, in good agreement with predictions. The mean gap is lower than the median of 2.52 eV, which reflects the presence of several ultra-low gap structures. The low gap and large variance mean that **167** may be able to absorb in the near IR. The redshifting is likely because of the six electron donating groups, which increase the HOMO energy, together with the crowding of the four *ortho* substituents. The latter distorts the molecule, leading to twisted configurations with smaller gaps. Finally, species **168** is more stable in *cis* form than *trans* form. The predicted *cis* stability is − 0.79 eV (−18 kcal/mol), in good agreement with the QC prediction of −0.92 eV (−21 kcal/mol). As mentioned above, this inverted stability can be a desirable property for photopharmacology.

To validate the yield results, we performed DANN-NAMD using highly accurate species-specific models. As described in Supplementary Note 13 B, we generated a model for each species by refining the base network with data from that species alone. The data was generated through several active learning cycles, resulting in 1200–2500 training geometries for each compound. We used this approach in place of ab initio NAMD because of the latter’s prohibitive cost for large molecules. The QC computational cost for fine-tuning was at most 3% of that of an ab initio simulation, and hence far less demanding. The average gradient calculation for a molecule with 50 atoms took 58 min for two surfaces using 8 cores, and the average NACV calculation took 55 min. Fine-tuning with 2000 geometries for a medium-sized molecule would thus take 30,000 core hours. For ab initio NAMD, a conservative estimate of 100 trajectories run for 1 ps each, with only one gradient computed per frame, would take 780,000 core hours.

We also computed the yields of *cis* and *trans* azobenzene for comparison. For these species we used full ab initio simulations, because of the central role of the unsubstituted compound as a reference point and because simulations were fairly affordable for such small molecules. Issues with spin contamination also hampered the fine-tuning process for these compounds (see Supplementary Note 13 B).

Initially we generated refined models for species **165**, **166**, **169** and **170**. It became clear early on that only **165** and **169** had high yields, and so we focused on those molecules thereafter. Using molecule-specific models, we computed the quantum yields of **165** and **169** to be 66 ± 1% and 37 ± 1%, respectively. The computed yields for *cis* and *trans* azobenzene are 60 ± 4% and 26 ± 3%, respectively, which are in excellent agreement with experiment^80^. Both of the new molecules have higher quantum yields than the associated base compounds. The improvement is particularly large for species **169**: its yield is 11 points higher than *trans* azobenzene, which is a relative enhancement of 42 percent. We show below that that this significant increase has an intuitive physical explanation.

**Fig. 5:**
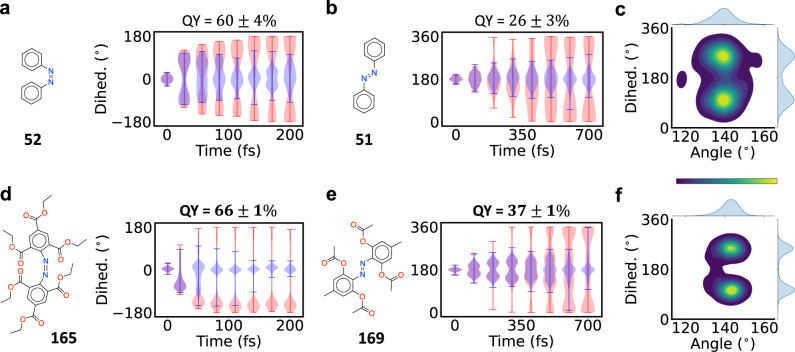
Visualization of high-accuracy non-adiabatic dynamics for several compounds of interest. **a**-**b**, **d**–**e** Violin plots showing the CNNC dihedral angle vs. time. Reactive and non-reactive NAMD trajectories are shown in red and blue, respectively. The violin width at a given dihedral angle indicates the density of trajectories with that angle. The yield of each compound is shown above the plots. For ease of visualization we have used the range [−180, 180] for *cis* dihedral angles and [0, 360] for *trans* dihedral angles. **c** Distribution of hopping geometries for *trans* azobenzene. **f** As in (**c**), but for the derivative **169**. The density is visualized with kernel density estimation as a function of the CNNC dihedral and $\max ({\alpha }_{{{{{{{{\rm{CNN}}}}}}}}},{\alpha }_{{{{{{{{\rm{NNC}}}}}}}}})$, where *α* is an angle. Yellow corresponds to the highest density and blue to the lowest. The marginal distributions over single coordinates are shown above and to the right of each plot.

The dynamics of the four molecules are shown in Fig. 5. Panels (a) and (b) show the CNNC dihedral angle vs. time for azobenzene, and panels (d) and (e) show the same for the derivatives. Both the substituted and unsubstituted *cis* isomers rapidly proceed through a rotational CI, but the derivative rotates much more quickly. Indeed, we see that the isomerization of the derivative is complete within 75 fs, while the base compound takes nearly 130 fs. The excited state lifetimes are 34.2 ± 0.3 fs and 63 ± 3 fs for the derivative and base compound, respectively, indicating that the former reaches the CI earlier than the latter. These observations may explain the enhanced yield, since a higher rotational velocity leads to more efficient isomerization^82^. We also note that the derivative rotates in only the counter-clockwise direction, while *cis* azobenzene rotates in both directions, but this is not expected to affect the yield.

The two *trans* molecules behave in qualitatively different ways. In *trans* azobenzene, the distribution of dihedral angles slowly widens with time (Fig. 5(b)). This is consistent with a rotational barrier^6,75^, as different trajectories overcome the barrier at different times, and so the torsion angle becomes uniformly distributed. Additionally, as seen in the marginal dihedral distribution of Fig. 5(c), many of the geometries hop near 180^∘^. This agrees with ref. ^6^, which identified a non-reactive planar CI and a reactive twisted CI as the main hopping points for *trans* azobenzene. The non-reactive CI leads exclusively back to *trans*, while the reactive CI leads to *cis* and *trans* in different proportions. Using the method described in Supplementary Note 13 C, we found that 26% of the trajectories proceed through the planar CI and 74% through the rotational CI. This is similar to the distribution reported in ref. ^75^. Approximately 36% of the rotational trajectories generate *cis* azobenzene, giving an overall yield of 26%. This is in good agreement with previous computational and experimental values^6^.

By contrast, nearly all trajectories of **169**, including non-reactive trajectories, rotate significantly. This can be seen in the marginal dihedral distribution in Fig. 5(f), in which the hops are tightly localized around 180 ± 77^∘^. Only 5% of the trajectories hop at the planar CI, which is five times lower than the base compound. Additionally, the yield of the rotational trajectories increases from 36 to 40%. The inhibition of the planar CI pathway, together with the enhancement of the rotational yield, leads to an overall yield increase from 26 to 37%. While the enhanced reactive yield does not have a simple explanation, the reason for the planar pathway inhibition can be clearly seen in Fig. 5(e). Whereas the rotation of **51** is stochastic, leading to a uniform distribution of angles, the rotation of **169** is initially concerted. Nearly all trajectories rotate in unison to a dihedral angle of 180 ± 45^∘^ at 300 fs. Past 300 fs, hopping begins and the trajectories separate from each other. Hence they proceed through the rotational reactive CI, and become distributed between 0^∘^ and 360^∘^ after hopping. The planar non-reactive CI is avoided because of the molecule’s initial rotation. This explanation is consistent with the presence of bulky *ortho* groups, which twist the equilibrium structure and hence weaken the N=N double bond. This lowers the excited state barrier to rotation, which leads to an initial torsion and hence increases the yield.

## Discussion

The DANN model shows high accuracy and transferability among azobenzene derivatives. One limitation is that the unseen species contained functional groups that were present to some degree in the training set. Model performance was generally higher for more highly represented functional groups, though some groups were highly represented yet difficult to fit, while others were weakly represented and well-fit (Supplementary Note 4). Moreover, the model cannot be applied to other chemical families without additional training data. Further, as shown in Supplementary Note 6, it substantially overestimates the excited state lifetime for a number of *trans* derivatives. On the other hand, semi-empirical methods provide qualitatively correct predictions across a variety of chemistries, but cannot match DANN’s in-domain accuracy, and cannot be improved with more reference data. Adding features from semi-empirical calculations, as done in the OrbNet model^83^, may therefore prove useful in the future. Recent developments accounting for non-local effects and spin states have improved neural network transferability^39^, and could also be beneficial for excited states. The model could be further improved with high-accuracy multi-reference calculations, solvent effects, and the inclusion of the bright *S*_2_ state. The use of spin-complete methods in particular is of crucial importance, since spin contamination prevented fine-tuning the model for the base compounds. It may also have affected the accuracy of the DANN model in general. Thus spin-complete, affordable alternatives are of particular interest^27^. Active learning could be accelerated through differentiable sampling with adversarial uncertainty attacks^84^, which would improve the excited state lifetimes. Transfer learning could also be used to improve performance for specific molecules. Only a small number of ab initio calculations would be required to fine-tune the model for an individual species.

Diabatization may also prove to be useful for reactive ground states. Reaction barriers can often be understood as transitions from one diabatic state to another^85^. The diabatic basis may make reactive surfaces easier to fit with neural networks.

In conclusion, we have introduced a diabatic multi-state neural network potential trained on over 630,000 geometries at the SF-TDDFT BHHLYP/6-31G* level of theory, covering over 8000 unique azobenzene molecules. We used DANN-NAMD to predict the isomerization quantum yields of derivatives outside the training set, and the results were correlated with experiment. We also identified several hypothetical compounds with high quantum yields, redshifted excitation energies, and inverted stabilities. The network architecture, diabatization approach, and chemical and configurational diversity of the training data allowed us to produce a robust and transferable potential. The model can be applied off-the-shelf to new molecules, producing results that approximate those of SF-TDDFT at orders of magnitude lower computational cost.

## Methods

### Network and training

As explained in Supplementary Methods A, a unique challenge for non-adiabatic simulations is their sensitivity to the energy difference between states. Using a typical neural network to predict energies and forces for NAMD leads to some molecules becoming incorrectly trapped in the excited state. This is partly caused by overestimation of the gap and/or an incorrectly shaped PES in the vicinity of the CI. To address this issue we introduce an architecture based on diabatic states, whose smooth variation leads to more accurate neural network fitting (Fig. 1(b)).

In general diabatic states must satisfy^86^

1 $${\left({{{{{{{{\bf{U}}}}}}}}}^{{{{\dagger}}} }\left[{\nabla }_{R}{{{\mathbf{H}}}}_{d}\right]{{{{{{{\bf{U}}}}}}}}\right)}_{nm}=\left\{\begin{array}{l}-{\overrightarrow{f}}_{n},{{{{{{{\rm{if}}}}}}}}\ n=m\\ {\overrightarrow{h}}_{nm},{{{{{{{\rm{if}}}}}}}}\ n\,\ne\, m.\end{array}\right.$$

where ∇_*R*_ is the gradient with respect to $\overrightarrow{R}$, **U** diagonalizes the diabatic Hamiltonian through

2 $${\left({{{{{{{{\bf{U}}}}}}}}}^{{{{\dagger}}} }{{{\mathbf{H}}}}_{d}{{{{{{{\bf{U}}}}}}}}\right)}_{nm}={E}_{n}{\delta }_{nm},$$

and ${\overrightarrow{f}}_{n}=-{\nabla }_{R}{E}_{n}$ is the adiabatic force for the *n*^th^ state. The dependence on $\overrightarrow{R}$ has been suppressed for ease of notation. ${\overrightarrow{h}}_{nm}$ is the force NACV,

3 $${\overrightarrow{h}}_{nm}(\overrightarrow{R})	=\left\langle {\psi }_{n}(\overrightarrow{r};\overrightarrow{R})\left|{\nabla }_{R}\hat{H}(\overrightarrow{r},\overrightarrow{R})\right|{\psi }_{m}(\overrightarrow{r};\overrightarrow{R})\right\rangle \\ 	=({E}_{m}(\overrightarrow{R})-{E}_{n}(\overrightarrow{R}))\,{\overrightarrow{k}}_{nm}(\overrightarrow{R}),$$

where $\hat{H}(\overrightarrow{r},\overrightarrow{R})$ is the clamped nucleus Hamiltonian, ${\psi }_{n}(\overrightarrow{r};\overrightarrow{R})$ is the *n*^th^ adiabatic wavefunction, and the matrix element is an integral over the electronic degrees of freedom $\overrightarrow{r}$. The vector ${\overrightarrow{k}}_{nm}(\overrightarrow{R})$ is the derivative coupling:

4 $${\overrightarrow{k}}_{nm}(\overrightarrow{R})=\left\langle {\psi }_{n}(\overrightarrow{r};\overrightarrow{R})\left|\right.{\nabla }_{R}{\psi }_{m}(\overrightarrow{r};\overrightarrow{R})\right\rangle$$

Combined with the following reference geometry conditions (Supplementary Methods C),

5 $$\begin{array}{l}({E}_{0},\,{E}_{1})=\left\{\begin{array}{ll}({d}_{00},\,{d}_{11}), &{{{{{{{\rm{if}}}}}}}}\overrightarrow{R}\in trans\\ ({d}_{22},\,{d}_{00}), &{{{{{{{\rm{if}}}}}}}}\overrightarrow{R}\in cis,\end{array}\right.\end{array}$$

we arrive at three sets of constraints, Eqs. (1), (2), and (5). In principle only Eqs. (1) and (2) are required for the states to be diabatic. However, we found the reference loss to provide a minor improvement in the gap near CIs (Supplementary Table I).

We use a neural network to map the nuclear positions ${\overrightarrow{R}}_{i}$ and charges *Z*_*i*_ to the diabatic matrix elements *d*_*nm*_, and a loss function to impose Eqs. (1), (2) and (5). The adiabatic energies *E*_*n*_ are generated by diagonalizing ${{{\mathbf{H}}}}_{d}$, and the forces and couplings by applying Eq. (1) and using automatic differentiation. The design of the network is shown schematically in Fig. 2(a). The general form of the diabatic loss function is

6 $${{{{{{{\mathcal{L}}}}}}}}={{{{{{{{\mathcal{L}}}}}}}}}_{{{{{{{{\rm{core}}}}}}}}}+{{{{{{{{\mathcal{L}}}}}}}}}_{{{{{{{{\rm{ref}}}}}}}}}+{{{{{{{{\mathcal{L}}}}}}}}}_{{{{{{{{\rm{nacv}}}}}}}}}.$$

Here ${{{{{{{{\mathcal{L}}}}}}}}}_{{{{{{{{\rm{core}}}}}}}}}$ penalizes errors in the adiabatic energies, forces, and gaps, ${{{{{{{{\mathcal{L}}}}}}}}}_{{{{{{{{\rm{ref}}}}}}}}}$ imposes Eq. (5) and ${{{{{{{{\mathcal{L}}}}}}}}}_{{{{{{{{\rm{nacv}}}}}}}}}$ imposes Eq. (1) for *n* ≠ *m*. The terms are defined explicitly in Supplementary Eqs. (1)–(3).

For the network itself we adopt the PaiNN equivariant architecture^38^. In this approach a set of scalar and vector features for each atom are iteratively updated through a series of convolutions (Fig. 2(a)). In the message block, the features of each atom gather information from atoms within a cutoff distance, using the interatomic displacements. The scalar and vector features for each atom are then mixed in the update phase. Hyperparameters can be found in Supplementary Table IV. Most were taken from ref. ^38^, but some were modified based on experiments with azobenzene geometries. Further details of the PaiNN model can be found in ref. ^38^. Once the elements of ${{{\mathbf{H}}}}_{d}$ are generated, the diabatic matrix is diagonalized to yield the transformation matrix **U** and the adiabatic energies *E*_*n*_. The vector quantities ${\overrightarrow{f}}_{n}$ and ${\overrightarrow{h}}_{nm}$ are given by Eq. (1). When non-adiabatic couplings are not required, the ${\overrightarrow{f}}_{n}$ can be calculated by directly differentiating the *E*_*n*_. This is more efficient than Eq. (1), since it requires only *M*_ad_ = 2 < *M*_d_(*M*_d_ + 1)/2 = 6 gradient calculations. This approach was used for NAMD runs, which required only diabatic energies, adiabatic energies, and adiabatic forces, while Eq. (1) was used for training.

### Data generation and active learning

Data was generated in two different ways. First, we searched the literature for azobenzene derivatives that had been synthesized and tested experimentally. This yielded 164 species (82 *cis* and 82 *trans*). For these species we performed ab initio NAMD, yielding geometries that densely sampled configurational space. Second, to enhance chemical diversity, we generated nearly 10,000 species through combinatorial azobenzene substitution. This was done using 48 common literature substituents and four common substitution patterns (Supplementary Tables X and XI). We then performed geometry optimizations, normal mode sampling, and inversion/rotation about the central N=N bond to generate configurations. QC calculations were performed on 25,212 combinatorial geometries. All calculations were performed with Q-Chem 5.3^87^, using SF-TDDFT^26^ with the BHHLYP functional^68^ and 6-31G* basis^67^.

Two neural networks were trained on the initial data and used to perform DANN-NAMD. Initial positions and velocities for DANN-NAMD were generated from classical MD with the Nosé-Hoover thermostat^88,89^. The initial trajectories were unstable because the networks had not been trained on high-energy configurations. To address this issue we used active learning^40,41^ to iteratively improve the network predictions (Fig. 2(b)). After each trajectory we performed new QC calculations on a sample of the generated geometries. For all but the last two rounds of active learning, geometries were selected according to the variance in predictions of two different networks, where the networks were initialized with different parameters and trained with different random batches. In the last two rounds, half the geometries were selected by network variance, and half by proximity to a CI. Further details are given in Supplementary Note 12. The new data was then added to the training set and used to retrain the networks. The cycle was repeated three times with all species and another two times with azobenzene alone. In all, we computed ground state gradients, excited state gradients, and NACVs with SF-TDDFT for 641,367 geometries. 96% of the geometries were from the 164 literature species. In total, 88% were generated through ab initio NAMD and 8% through active learning. The remaining 4% were from the combinatorial species. 1.5% were generated through geometry optimizations, 1.5% through inversion/rotation, and 1% through normal-mode sampling.

We initially set out to train a model using energies and forces alone. Since analytic NACVs are unavailable for many ab initio methods, an adiabatic architecture could have been used with a wider variety of methods. NACVs also add computational overhead, and so generating training data for an adiabatic model would have taken less time. To this end we initially used the Zhu-Nakamura (ZN) surface hopping method^82^, which only requires adiabatic energies and forces. However, the issues with adiabatic models described in Supplementary Note 6 led us to develop the diabatic approach. Since diabatic states can be used with any surface hopping method, we used the diabatic model to perform Tully’s fewest switches (FS) surface hopping^61^ after the last round of active learning. All results in the main text use the FS method. A comparison of FS and ZN results is given in Supplementary Note 6.

## Supplementary information

Supplementary Information

Description of Additional Supplementary Files

Supplementary Data 1

Supplementary Data 2

Supplementary Data 3

## Data Availability

The quantum chemistry data generated in this study has been deposited in the Materials Data Facility database at 10.18126/unc8-336t. A detailed description of how to load and interpret the data is given in the README file. Source data of experimental and predicted quantum yields are provided in the Supplementary Information/Source Data file.

## Code availability

Trained models and computer code are available in the Neural Force Field repository at https://github.com/learningmatter-mit/NeuralForceField. Notebook tutorials explain how to train the models and perform DANN-NAMD.
