# Supplementary material for: Excited state non-adiabatic dynamics of large photoswitchable molecules using a chemically transferable machine learning potential
Source: Nat Commun. 2022 Jun 15;13:3440. doi: 10.1038/s41467-022-30999-w (PMC9200747; doi:10.1038/s41467-022-30999-w)
Supplement: Supplementary file 1 — Supplementary Information [file 41467_2022_30999_MOESM1_ESM.pdf]

Supplementary Information
Excited state, non-adiabatic dynamics of large photoswitchable molecules using a chemically transferable machine learning potential

S. Axelrod *et al.*

SUPPLEMENTARY METHODS

A. Relevance of adiabatic gap

Accurate prediction of the energy gap is crucial for generating reliable NAMD results. The gap controls the hopping rate, and in turn observables like the photoisomerization quantum yield, excited state lifetime and time-resolved photoelectron spectrum [1]. To understand the importance of the gap, consider that in most approaches, the hopping rate is determined by the derivative form of the NACV [2, 3]. The derivative coupling between states n and m can be written as $\vec{h}_{nm}/\Delta E_{nm}$, where ΔE_{nm} is the energy gap and $\vec{h}_{nm}$ is the force coupling [4, 5]. The gap in the denominator leads to singular derivative coupling at CIs, and therefore to a guaranteed hop. The coupling can alternatively be obtained from the gap alone from through its first and second derivatives [6]. The energy difference also features prominently in the Zhu-Nakamura method, in which the hopping rate is approximately exponential in the square of the gap [7]. Therefore, it is crucial to accurately predict the energy gap in any NAMD simulation.

B. Diabatization

The adiabatic energies of a system, $\{E_n(\vec{R})\}$, are the energies produced by a QC calculation. They depend on the nuclear coordinates $\vec{R}$, and form the usual Born-Oppenheimer PESs. In the adiabatic basis the nuclear kinetic energy operator, related to ∇^2_R, is non-diagonal. Its off-diagonal elements are related to the NACVs, which generate transitions between adiabatic PESs [8]. The derivative form of the NACV also becomes singular at CIs, which is an undesirable numerical property. The diabatic basis is designed to remove this singularity [9]. The diabatic Hamiltonian $\mathbf{H}_d(\vec{R})$ is a rotation of the adiabatic energies into a new basis, given by $\mathbf{H}_d(\vec{R}) = \mathbf{U}(\vec{R})\,\mathrm{diag}(\{E_n(\vec{R})\})\,\mathbf{U}^\dagger(\vec{R})$. Here diag denotes a diagonal matrix, and $\mathbf{U}(\vec{R})$ is a rotation matrix that depends on the nuclear coordinates (see Eq. (2) in the main text). $\mathbf{H}_d$ can also be viewed as the clamped nucleus Hamiltonian expressed in the basis of diabatic wave functions. These wave functions are given by $\psi_{\mathrm{d},n}(\vec{r};\vec{R}) = \sum_m U_{nm}(\vec{R})\,\psi_{\mathrm{ad},m}(\vec{r};\vec{R})$, where $\psi_{\mathrm{ad},m}$ is the mth adiabatic wave function and $\vec{r}$ denotes the electronic coordinates.

The diabatic states are defined such that the nuclear kinetic energy is approximately diagonal [9]. The states are instead coupled through off-diagonal elements in the potential energy matrix, known as diabatic couplings. The diabatic couplings are smooth functions of the nuclear coordinates. The diagonal elements are also smooth, maintaining their orbital character and switching energy ordering through a CI (Fig. 1(b) in the main text). In many applications, diabatic states are preferred over adiabatic ones because the singular coupling is removed. Here we prefer them because diabatic energies are easier to fit than adiabatic energies. That is, even if one is only interested in adiabatic energies and not NACVs, it is easier to learn the diabatic energies and then diagonalize $\mathbf{H}_d$ than to learn the adiabatic energies directly. This is because the diabatic states possess no CI cusps or avoided crossings (Fig. 1 in the main text), and are thus more easily fit by interpolating functions such as neural networks. As discussed below, diabatic states improve the model accuracy for species outside the training set.

While many diabatization methods exist, the most common ones cannot be straightforwardly applied to the current problem. Property-based methods were developed for charge-transfer type problems [10], while orbital-based approaches [11] are not implemented for TDDFT. Diabatic models that are parameterized by electronic structure

data [4, 12, 13] are not designed for systems undergoing large distortions. Approaches that solve for the adiabatic-to-diabatic transformation matrix [14] are difficult to implement in practice, because the matrix varies rapidly near a CI.

Recent work introduced neural network diabatization based on reference geometries [15, 16]. In this procedure one assumes that the diabatic Hamiltonian is diagonal at a set of known reference geometries. At such geometries the elements of $\mathbf{H}_d$ are equal to the adiabatic energies, but possibly reordered. For example, for two states and two reference geometries, one would have $\mathbf{H}_d = \mathrm{diag}(E_0, E_1)$ at the first geometry and $\mathbf{H}_d = \mathrm{diag}(E_1, E_0)$ at the second (Fig. 1(b) in the main text). A neural network is then trained to produce $\mathbf{H}_d$, such that its eigenvalues are always equal to the adiabatic energies, and its elements are as above at the reference geometries. These two constraints generate $\mathbf{H}_d$ at all intermediate geometries.

This method was successfully applied to thiophenol dissociation, yielding results consistent with the fourfold way [15]. However, as shown by the NACV error in Supplementary Table I, this method alone does not generate true diabatic states for azobenzene. To understand why, consider that near a CI, the true diabatic coupling d_{01} must closely resemble the coupling shown in Fig. 1(b) in the main text (d_{nm} is shorthand for $(\mathbf{H}_d)_{nm}$). This is because d_{01} must be linear in displacements about a CI, and quadratic only for Renner–Teller type intersections [17]. However, for two diabatic states, only the square of the diabatic coupling $|d_{01}|^2$ enters into the expression for the adiabatic energies. The model might then generate an off-diagonal element similar to $|d_{01}|$. This error would incur no penalty on the network, because the diagonal elements would properly switch ordering and the adiabatic energies would be correct. Hence the model would not generate smooth diabatic states.

To remedy this issue we used the combined loss described in the Methods section. The NACV component of this loss was also used in Ref. [18]. We note that the number of diabatic states and choice of orderings in Eq. (5) in the main text may depend on the system. For azobenzene we were interested in fitting $M_{\mathrm{ad}} = 2$ adiabatic states, and in general one should use $M_{\mathrm{d}} \geq M_{\mathrm{ad}}$ diabatic states. In this work chose $M_{\mathrm{d}} = 3$ because it significantly improved on the results of $M_{\mathrm{d}} = 2$. In fact, with $M_{\mathrm{d}} = 2$, the R^2 correlation of the force NACV was negative. The orderings in Eq. (5) in the main text were chosen by taking a small sample of azobenzene configurations, training several small models with different diabatic orderings, and picking the one with the best results. Thus no system knowledge is required to choose the orderings. The only system knowledge required is the set of reference geometries[1], but the reference loss can be omitted with negligible impact on model performance.

The diabatic model leads to true CIs, where the ground and excited state energies are exactly equal. To see why, consider Fig. 3(b) in the main text, which shows the two diagonal diabatic elements (red and blue) and the off-diagonal coupling (light gray). The off-diagonal element passes linearly through zero in the $\vec{h}$ direction. The diagonal elements cross in the $\vec{g}$ direction, and so $\Delta \equiv d_{11} - d_{00}$ passes linearly through zero. Therefore, one can begin at a geometry for which $\Delta = 0$, and move in the $\vec{h}$ direction until $d_{01} = 0$ without changing Δ. The final geometry will therefore be a CI. The fact that both d_{01} and Δ are locally linear, and hence must pass through zero, is known theoretically [17] and properly predicted by the model.

---

[1] Typically the reference geometries are reactants and products. For reactions in which the product is not known *a priori*, one might use the following approach. First, train an adiabatic network on a single molecule. We have found that adiabatic models match or outperform diabatic ones for single species. Then use the model to simulate dynamics. By using active learning to improve the model's coverage of configurational space, the simulations can eventually discover the product. If derivatives of the molecule are expected to have similar reactions, then their products are also now known. With knowledge of the reactants and products, and hence the reference geometries, one can now build a diabatic model. If this is not possible, then one can train the model without $\mathcal{L}_{\mathrm{ref}}$. Supplementary Table I shows that this will likely decrease the model performance near CIs by a small amount.

C. Network loss

The neural network loss terms are defined as:

$$\begin{aligned}\mathcal{L}_{\mathrm{core}} &= \sum_n \rho_{E_n} \cdot \mathrm{mse}\big(E_n\big) + \rho_{f_n} \cdot \mathrm{mse}\big(\vec{f}_n\big) \\ &\quad + \sum_{n>m} \rho_{\Delta E_{nm}} \cdot \mathrm{mse}\big(\Delta E_{nm}\big) \qquad (1)\\ \mathcal{L}_{\mathrm{ref}} &= \sum_n \rho_{\mathrm{ref}} \cdot \mathrm{mse}\big(d_{nn}(\vec{R})\big)\big|_{\vec{R}\in\{cis,\ trans\}} \qquad (2)\\ \mathcal{L}_{\mathrm{nacv}} &= \sum_{n>m} \rho_{\mathrm{nacv}} \cdot \mathrm{mse}\big(A_n(\vec{R})A_m(\vec{R})\ \vec{h}_{nm}(\vec{R})\big), \qquad (3)\end{aligned}$$

where $\Delta E_{nm} = E_n - E_m$ is the energy gap. Each loss function is a sum over mean squared error (MSE) terms scaled by different weights ρ. For scalar molecular quantities X the loss is given by $\mathrm{MSE}(X) = \sum_{j=1}^{M}(X_j - \hat{X}_j)^2/M$, where $\hat{X}$ is the predicted quantity and the sum is over M geometries in a batch. For atomic vectorial quantities the mean is additionally over the $3L_j$ vector components, where L_j is the number of atoms in the jth geometry.

The loss term $\mathcal{L}_{\mathrm{core}}$ contains the usual energy and force losses, plus an additional term for gap errors. While the gap term was not used in previous work, we found it to be crucial for the systems studied here. Indeed, without this loss term, one would expect the gap MAE in adiabatic models to be the geometric sum of the energy MAEs. This is what we found when using $\mathcal{L}_{\mathrm{core}}$ without the gap loss. Supplementary Table I shows that, when using the full core loss, the gap MAE is actually lower than each individual energy MAE.

The reference loss, $\mathcal{L}_{\mathrm{ref}}$, is a sum over geometries $\vec{R}$ which are considered to be *cis* or *trans*. At these geometries the target d_{nn} are given by Eq. (5) in the main text. A geometry is considered to be *cis* or *trans* if its central CNNC atoms deviate from those of the equilibrium structure by <0.15 Å. The distance is computed as the root-mean-square deviation (RMSD) among the atoms after alignment.

The NACV loss imposes diabaticity. It involves the force NACV $\vec{h}_{nm}$, and a phase correction $A_n(\vec{R}) = \pm 1$. The phase correction is chosen separately for each geometry to minimize the error between the predicted and target force coupling. This factor is necessary because each adiabatic wavefunction can have an arbitrary sign [19]. The signs cancel for diagonal terms like E_n and $\vec{f}_n$, but not for off-diagonal terms like $\vec{h}_{nm}$. The A_n account for these arbitrary sign changes.

D. Data generation

To train a useful model one must generate reliable QC data. TDDFT [20] typically offers a good compromise between speed and accuracy for modeling excited states. However, it has known instabilities near CIs [21], and, as a result of the Brillouin theorem, produces the wrong branching space dimensionality for S_0/S_1 intersections [22]. These issues, which can be traced back to the single-reference description of the excitation, can be partially alleviated with SF-TDDFT [23]. In this approach the excitation is performed with respect to a high-spin reference state. This yields some transitions that have double-excitation character with respect to the singlet ground state. Here we used SF-TDDFT with the BHHLYP functional [24] and the 6-31G* basis [25]. SF-TDDFT/BHHLYP is well-benchmarked for CIs in a number of molecules [26, 27]. Because SF is not spin complete, the singlet states must be identified based on their square spins $\langle S^2 \rangle$. We used the approach of Ref. [28], which identifies singlets as the two states with the lowest $\langle S^2 \rangle$ from the three excitations of lowest energy. Calculations were performed with the Q-Chem package [29].

Near-CI regions of the combinatorial species were sampled by setting the central CNNC dihedral angle of relaxed geometries to ±90 degrees and/or the CNN/NNC angles to 180 degrees. The other internal coordinates were not changed. The former corresponds to the rotation pathway and the latter to inversion. This led to 11 possible combinations of rotation and inversion, including pure rotation, pure inversion, concerted inversion, and inversion-assisted rotation [30].

SUPPLEMENTARY NOTES

Supplementary Note 1. Model accuracy and ablation studies

	E_0	E_1	ΔE_{01}	$(\Delta E)_{\text{small}}$	$\text{sgn}(\Delta E)_{\text{small}}$	$\vec{F}_0$	$\vec{F}_1$	$\vec{h}_{01}$
DANN	3.06	3.77	1.89	**0.97**	-0.29	1.72	2.31	**1.36**
$-\mathcal{L}_{\text{nacv}}$	2.31	**2.49**	**1.65**	1.22	0.52	1.72	2.39	2.21
$-\mathcal{L}_{\text{ref}}$	2.21	2.58	1.68	1.06	0.16	1.72	2.32	1.37
adiabat	**2.11**	3.24	2.43	1.24	0.60	1.69	2.31	—
adiabat (st. 1)	3.28	2.99	1.88	1.49	0.95	**1.67**	**2.30**	—
median	16.86	19.04	22.80	36.04	36.04	17.27	17.18	2.05

Supplementary Table I. Performance of different ablated models. Each column, apart from $\text{sgn}(\Delta E)_{\text{small}}$, shows the MAE of a different quantity. $\text{sgn}(\Delta E)_{\text{small}}$ is the mean signed error of $(\Delta E)_{\text{small}}$, given by $\text{mean}\{(E)^{\text{pred}}_{\text{small}} - (\Delta E)^{\text{target}}_{\text{small}}\}$. "st. 1" indicates the first stage of training of the adiabatic model, as described in Supplementary Note 8. The best score in each category is shown in bold.

Here we compare the DANN model to a model trained without a NACV loss ("$-\mathcal{L}_{\text{nacv}}$"), a model trained without a reference geometry loss ("$-\mathcal{L}_{\text{ref}}$"), an adiabatic model, and a median predictor. The latter predicts the median ground- and excited-state energy for each species, and the median value among all species for other quantities. The MAEs for unseen species are compared in Supplementary Table I. Half the geometries were sampled randomly and half by proximity to a CI, as described in Supplementary Note 8. Of particular interest are $(\Delta E)_{\text{small}}$, the MAE of the gap when it is under 0.2 eV, and $\text{sgn}(\Delta E)_{\text{small}}$, the average overestimation of small gaps by the model. DANN actually underestimates the small gaps, indicating that it does not "miss" conical intersections. Using an adiabatic model or removing the NACV loss increases both the error and overestimation of $(\Delta E)_{\text{small}}$. The same is true of removing $\mathcal{L}_{\text{ref}}$, but the effect is smaller. While the changes in $(\Delta E)_{\text{small}}$ appear minor, we show in Supplementary Note 6 that they lead to noticeable quantum yield differences for a number of species.

Also of note is the difference in NACV error among the different models. Removing $\mathcal{L}_{\text{nacv}}$ substantially increases the NACV error, even when $\mathcal{L}_{\text{ref}}$ is used. Since $\mathcal{L}_{\text{nacv}}$ imposes diabaticity, this shows that the reference loss alone does not give accurate diabatic states. We also see that the adiabatic model is much worse at predicting ΔE away from the CI than the diabatic model. This is because of the large loss weight used for $(\Delta E)_{\text{small}}$ in the second stage of adiabatic training (see Supplementary Note 8). Indeed, the results after the first stage are much better for ΔE, but far worse for $(\Delta E)_{\text{small}}$. Finally, the models without reference or NACV losses have far lower energy errors than DANN. It may be possible to decrease DANN's energy errors by increasing the weight of the energy loss.

A key benefit of the diabatic model is that it accurately predicts the gap near CIs. It also produces the NACVs, adiabatic energies, and forces all with one model. Without the diabatic model, one might learn the force coupling as a separate property. This has been done in other work by taking the gradient of a learnable scalar [6, 31]. However, one would still have to divide by the adiabatic gap to obtain the derivative coupling. Thus gap prediction errors from an adiabatic model would still be problematic. A separate option would be to compute the NACV through the Hessian of the gap [6]. This is quite slow, and would have similar problems with the gap.

Supplementary Table II shows the test set statistics for the DANN model trained on all species. This model was used for virtual screening, as described in the main text. Here the test set simply consists of 5,000 held-out geometries. Unlike in Supplementary Table I, the test set contains mostly seen species, and the geometries were not generated from DANN-NAMD with the trained model. The results are quite similar to the "seen species" rows in Table I in the main text.

Supplementary Note 2. Experimental data

We performed an extensive literature search to find quantum yields of azobenzene derivatives. To best compare to the vacuum conditions simulated here, we chose quantum yields measured in non-polar solvents. When multiple results

	E_0	E_1	ΔE_{01}	$(\Delta E_{01})_{\text{small}}$	$\vec{F}_0$	$\vec{F}_1$	$\vec{h}_{01}$
MAE (↓)	0.75	0.96	0.72	0.40	0.94	1.13	0.87
R^2 (↑)	1.00	0.99	0.99	0.97	1.00	0.99	0.88

Supplementary Table II. Test set MAE for the DANN model trained on all species.

were available in different non-polar solvents, we computed the prediction error relative to the mean experimental value. To compare to the simulated S_1 dynamics, we selected yields measured at the peak of the highest-wavelength absorption band, which is a dipole-forbidden $n-\pi^*$ transition in unsubstituted azobenzene. All sources used here specifically reported yields at wavelengths close to the $n-\pi^*$ and $\pi-\pi^*$ absorption peaks, so it was straightforward to choose the appropriate wavelength. Lastly, we only selected yields at or around room temperature, since yields can have a strong temperature dependence [32].

Supplementary Note 3. Sources of error

A. *Experimental error*

There are two main sources of uncertainty in the experimental quantum yield results. The first is the error in the absorption coefficients ε of the two isomers. The absorption strengths are used in standard rate equations to compute the yield; see, for example, Refs. [33, 34] and the supplementary of Refs. [35, 36]. The error can be traced back to the overlapping absorption spectra of the two isomers, which must be disentangled. The magnitude of the error depends on the method used to compute ε, which itself is related to the year of publication. For example, Ref. [33], published in 1988, indicated that the absorption coefficients were the primary source of error, but did not give an estimate for their uncertainty. They isolated the different isomers using chromatography and computed their absorption coefficients separately. When the isomers could not be isolated in sufficient amounts, the authors used the time-dependent absorption approach of Ref. [37]. This method had relative errors of of ±25% for $n-\pi^*$ quantum yields [37] (i.e, yield → yield × (1 ± 0.25)). Ref. [34], published in 2004, reported a yield of 0.7 to 1.0 for compound **20**, a large range stemming from the overlap of the isomers' spectra and the method used to separate them [38]. More modern methods have lower errors; for example, Ref. [36], published in 2015, reported a relative error of only ±10% in the supplementary.

The second important source of error is the overlap of the $n-\pi^*$ and $\pi-\pi^*$ absorption bands. The more strongly the two bands overlap, the higher the error of using only the S_1 state in the dynamics. Moreover, many experiments do not irradiate at the precise maximum of the $n-\pi^*$ band. Often a representative $n-\pi^*$ or $\pi-\pi^*$ wavelength is chosen, and then used for each derivative [32, 35], even though the derivatives have different absorption peaks.

To get an idea of the range of errors this could introduce, consider the results of Ref. [39] from 1958. This work computed the quantum yield of unsubstituted azobenzene over several wavelengths. The *trans* to *cis* yield was measured as 21% at 405 nm and 27% at 436 nm (10^{-3} M solution). The difference is because 405 nm light excites more of the $\pi-\pi^*$ band (313 nm) than 436 nm light. 405 nm excitation leads to a lower yield because the $\pi-\pi^*$ yield is only 11%. The difference is even more pronounced for the *cis* quantum yield, which drops from 55% to 40% moving from 436 nm to 546 nm. This result does not have a simple explanation, as there is no lower energy band beyond 436 nm. In principle, the error due to overlapping bands could be mitigated with an explicit dipole-electric field coupling term [40], which would excite multiple states in different proportions. For derivatives with completely overlapping $n-\pi*$ and $\pi-\pi*$ bands, the S_1 approximation would incur a maximum error of the S_2 yield minus the S_1 yield. This is because the S_2 state is much brighter than S_1, and would therefore dominate the experimental yield. The yield difference is about 10 percentage points for both *cis* and *trans* unsubstituted azobenzene [41, 42]. Therefore, in the worst case scenario, we would expect an error of about 10 percentage points from the S_1 approximation. The error would likely be closer to the 6% reported in Ref. [39] at wavelengths of moderate overlap.

We can further quantify these errors by computing the range of results from different studies. Measurements of the $t \to c$ azobenzene yield range from 20% to 28% between 1979 and 1987 [37, 41, 43], though this could also be related to solvent effects (Supplementary Note 3 B).The yields of compounds **9** and **10** range from 16% to 24% and 44% to

50%, respectively, with references from 1962 and 1988 [32, 33]. Aggregating the results of all three compounds gives an average yield range of 7.3 percentage points, and an average relative error of ±14%.

A final source of uncertainty is specific to Refs. [44, 45]. Several species were reported to have zero yield over a large range of irradiation wavelengths. However, the dipole-allowed S_2 transition was highly redshifted and thus overlapped strongly with the S_1 transition. The absorbance changes after irradiation were quite small, indicating a near-zero yield, but could have been due to the S_1 transition. Hence it is possible that the S_1 yield is not precisely zero.

B. *Computational error*

There are several sources of computational error. The first is error in the PES, which can be decomposed into error from SF-TDDFT and error from the model. While it is intractable to perform SF-TDDFT for all species with experimental data, our results for unsubstituted azobenzene suggest that it is rather accurate. As reported in the main text, we computed yields of 60 ± 4% and 26 ± 3% for $c \rightarrow t$ and $t \rightarrow c$, respectively. Experimental measurements between 1979 to 1987 gave $t \rightarrow c$ yields between 20% and 28% [37, 41–43]. Experimental measurements in 1974 and 1979 gave $c \rightarrow t$ yields of 55% and 56% [41, 42], while a measurement in 1958 gave 48% ± 5% [39]. All measurements were performed in non-polar solvents. The yields computed with the original model were 59% and 37% for $c \rightarrow t$ and $t \rightarrow c$, respectively. Hence the main source of error seems to be the model, rather than SF-TDDFT.

Solvent effects may also affect the yield. These effects can be decomposed into a systematic term and a non-systematic term. It has been argued that non-polar solvents systematically reduce the quantum yield relative to vacuum [46]. However, careful analysis of the experimental data reported in [47] and cited in [46] does not support this conclusion. Indeed, given the good agreement between non-polar experimental results and both SF-TDDFT and hh-TDA DFT [48], systematic effects of non-polar solvents are likely to be small. Solvent-specific effects are part of the range of reported experimental values, since different works often use different non-polar solvents. Typical ranges were discussed in Supplementary Note 3 A.

A final source of error is the approximations in surface hopping. A large body of literature has examined surface hopping's accuracy; see, for example, Refs. [49–52]. In this work, we can roughly evaluate its performance by comparing its results to those of *ab initio* multiple spawning (AIMS) [48]. AIMS is a fully quantum mechanical method and can thus be treated as a benchmark. We computed the $t \rightarrow c$ yield as 26 ± 3% using surface hopping with SF-TDDFT, and Ref. [48] computed the yield as 24% ± 6% using AIMS with hh-TDA DFT. While there may be some error cancellation from the different quantum chemistry methods, the good agreement is still encouraging.

Supplementary Note 4. Influence of different functional groups

Here we discuss how the model transferability depends on the functional groups in a compound. To answer this question, we analyzed each of the 40 unseen species in the test set, and computed the model error for each geometry. As described in the main text, the geometries were taken from DANN-NAMD with the trained model; half were selected by proximity to a CI and half selected randomly. For each functional group, we aggregated the errors of all geometries that contained the group, and then computed the mean.

The functional groups with the lowest gap errors are shown in Supplementary Fig. 1(a), and those with the highest are shown in panel (b). Supplementary Table III shows the number of times that each functional group appears in the training set, together with the errors for all properties. Of the groups with the lowest errors, we see that both **B** and **C** are well-represented in the training set, which may explain their accurate results. The other groups each have about 1,000 samples in the training set. This is smaller than that of **B** and **C**, but not negligible. The compounds are also rather simple, which may partly explain their low error.

Of the groups with the highest gap error, only the nitro group **G** is well-represented in the training set. There are almost 10,000 training geometries with nitro groups, yet the error is still quite high. This may be because NO_2 is a strong electron-withdrawing group, and can thus have a significant effect on the electronic structure of azobenzene. Of the remaining substituents, both **H** and **J** are rather complicated, and each has only about 200 samples in the training set. Groups **F** and **I** both have about 1,000 samples in the training set, and are thus moderately represented. **I** may have large errors because of the electron-withdrawing effects of the three fluorines, or because it is found together

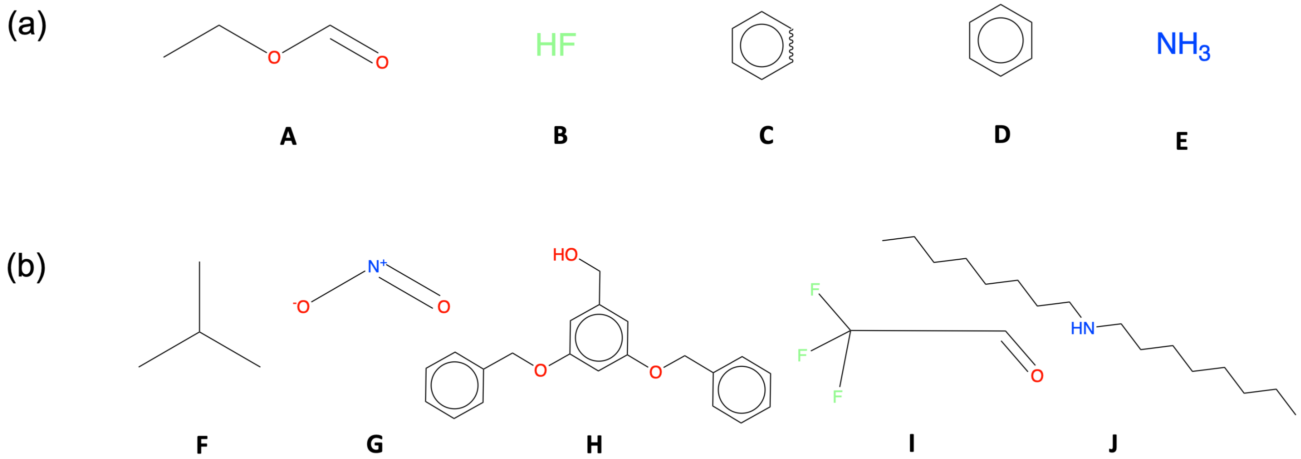

Supplementary Figure 1. Functional groups in the test set of unseen species. (a) The five functional groups with the lowest gap error. Error increases from left to right. **C** is fused to a benzene ring in azobenzene. (b) The five groups with the highest gap error. Error decreases from left to right.

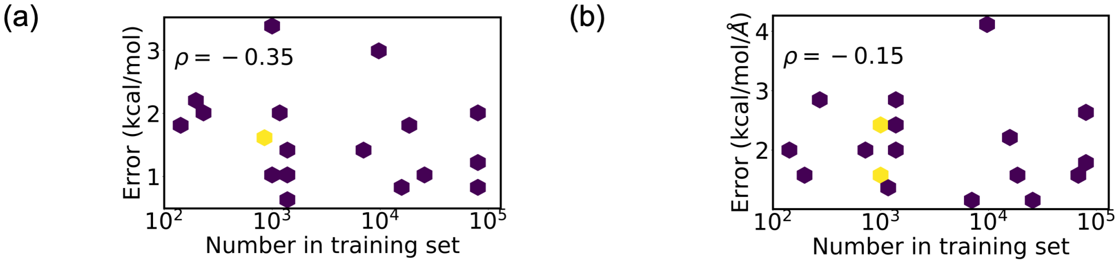

Supplementary Figure 2. Test set error by functional group, plotted against the number of times the group appears in the training set. The Spearman rank correlation ρ is also shown. (a) Gap error. (b) Excited state force error.

with **J** in compound **20**. **F**, the *tert*-butyl group, likely has large errors simply because it is bulky and thus leads to distorted geometries.

We see that the transferability of the model depends on how well-represented a functional group is in the training set, and how complicated its electronic effects are. The former is supported by Supplementary Fig. 2, which shows that the functional group error is anti-correlated with its prevalence in the training set. However, the effect is stronger for the gap than the excited state forces ($\rho = -0.35$ and $\rho = -0.15$, respectively), and neither fully explains the error. The remaining error can best be explained by analyzing the groups themselves.

	Num. train	E_0	E_1	ΔE_{01}	$(\Delta E)_{\text{small}}$	$\text{sgn}(\Delta E)_{\text{small}}$	$\vec{F}_0$	$\vec{F}_1$	$\vec{h}_{01}$
**A**	1,219	1.05	0.94	0.63	0.61	-0.04	1.24	1.32	1.19
**B**	71,940	0.95	1.02	0.81	0.52	-0.01	1.33	1.66	1.62
**C**	17,624	1.08	1.14	0.85	0.37	0.12	1.24	1.52	1.62
**D**	994	0.73	1.00	0.91	0.44	-0.07	1.18	1.70	1.54
**E**	1,154	1.09	1.21	0.95	0.72	-0.04	1.61	2.42	1.41
**F**	979	2.28	3.36	3.39	1.14	-0.31	1.53	1.53	1.96
**G**	9,578	0.94	3.49	2.90	0.68	-0.16	1.59	4.11	1.83
**H**	200	1.62	3.17	2.18	0.44	-0.13	0.99	1.67	0.68
**I**	1,225	1.33	2.90	2.10	0.78	-0.17	1.63	2.90	0.91
**J**	236	1.33	2.90	2.10	0.78	-0.17	1.63	2.90	0.91

Supplementary Table III. Test set error by functional group. The groups are divided into those with the lowest gap errors (top) and those with the highest (bottom). "Num. train" refers to the number of geometries in the training set that contain the functional group. While each of the functional groups is contained in the training set, the precise combinations and arrangements of groups (and hence the molecular graphs) are unique to the test set.

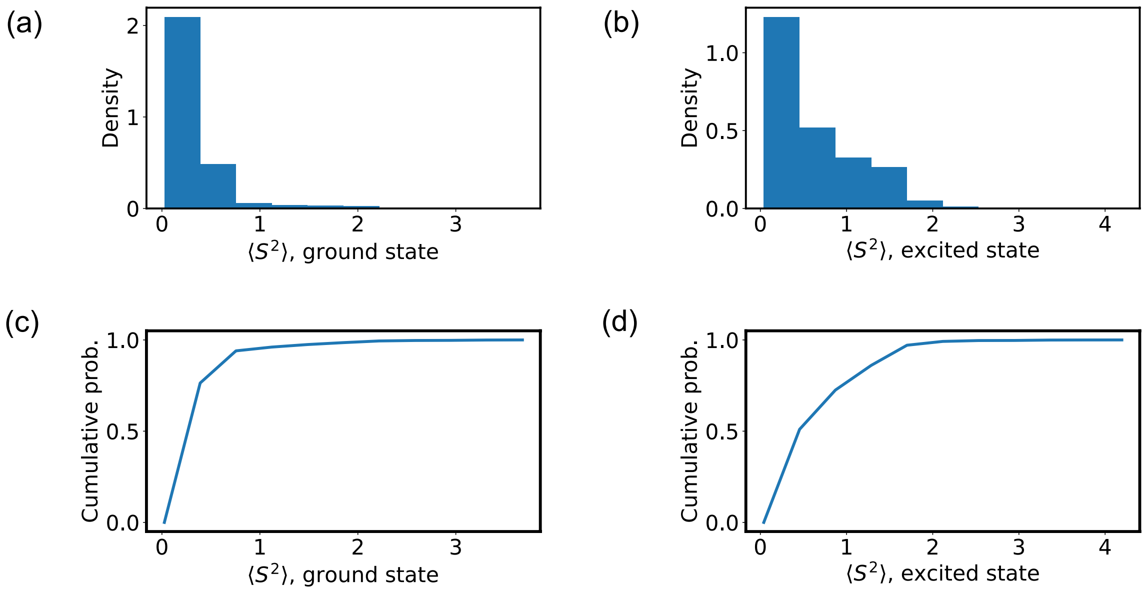

Supplementary Figure 3. Analysis of spin contamination in the training set. (a) Distribution of $\langle S^2 \rangle$ in the ground state. (b) As in (a), but for the excited state. (c) Cumulative probability as a function of $\langle S^2 \rangle$ in the ground state. (d) As in (c), but for the excited state.

Supplementary Note 5. Spin contamination

Here we discuss the amount of spin contamination in the training set. Supplementary Fig. 3 shows the square spin, $\langle S^2 \rangle$, for both the ground and excited states. The spin contamination is quite low for the ground state, with $\langle S^2 \rangle$ under 1.0 for 96% of geometries. It is much higher for the excited state, with 76%, 82%, and 93% of geometries having $\langle S^2 \rangle$ under 1.0, 1.2, and 1.4, respectively. Most of the geometries with excited-state $\langle S^2 \rangle \gtrapprox 1.2$ were near the non-reactive S_1/S_2 CI, characterized by near-planarity and CNN angles near 108° [48]. The S_0/S_1 CIs, on the other hand, did not have severe spin contamination. The spin-contamination near the S_1/S_2 CI led to difficulty in

Hyperparameter	Meaning	Value or name
F	dimension of hidden atomic features	128
n_{conv}	number of convolutions	5
n_{RBF}	number of radial basis functions (RBF)	20
R_{cut}	cutoff distance for convolutions	5.0 Å
activation function	activation used in message-passing and readout	Swish
learnable k	whether k parameters in RBF are learnable	true
skip	output is sum of per-convolution outputs	true only for adiabatic
M_{d}	number of diabatic states	3
M_{ad}	number of ground truth adiabatic states	2

Supplementary Table IV. Model hyperparameters

fine-tuning the model for unsubstituted azobenzene. We also visually inspected a sample of geometries with extreme spin contamination, $\langle S^2 \rangle > 1.8$, and found that nearly all had broken apart. We kept these geometries in the training set so that the model could learn the high energy of bond breaking.

Supplementary Note 6. Surface hopping results with different models

Supplementary Fig. 4 compares ZN hopping statistics of adiabatic and diabatic models for unseen species. Panel (a) shows the distribution of hopping percentages among species. The hopping percentage is defined as the percent of trajectories for a given species that end in the ground state. The y-axis is the percent of all species that correspond to each bin. Panel (b) has an analogous plot, but with the hopping percentage replaced by the lifetime. The lifetime was estimated from an exponential fit of S_1 population, $p = \exp(-(t - t_{\mathrm{on}})/\tau)\ \Theta(t - t_{\mathrm{on}}) + \Theta(t_{\mathrm{on}} - t)$. Here $p \in [0, 1]$ is the S_1 population, t is the time, t_{on} is the fitted turn-on time, τ is the fitted lifetime, and Θ is the Heaviside step function. Trajectories that did not contain any hops were assigned the maximum lifetime of all the other trajectories.

The *cis* derivatives have lifetimes around 50 fs, consistent with computational results for *cis* azobenzene [28]. Nearly 100% of all *cis* trajectories ended in the ground state. The *trans* derivatives have a wide distribution of lifetimes. Some are between 1 and 2 ps, which is similar to *trans* azobenzene [28]. Others are in the range of tens to hundreds of ps, which reflects the high proportion of trajectories that never returned to the ground state. These are very likely incorrect. They may be because of barriers between S_1 minima and S_0/S_1 CIs, which are known to exist for *trans* azobenzene [48]. Relatively small errors in the barrier may lead to large over-estimations of the lifetime.

Excited state barriers make it even more important to have accurate PESs near CIs. Since trajectories spend little time near crossing regions, trapping becomes even more severe when the gap near CIs is overestimated. The diabatic model helps to address this problem: in each plot we can see that the diabatic model leads to more hopping. For example, using the adiabatic model, 21% of species have hopping percentages under 10%. This number is reduced to 13% for the diabatic model.

The diabatic model also improves the quantum yield. Supplementary Fig. 5 shows the ZN quantum yields with the diabatic model, and Supplementary Fig. 7 shows the diabatic FS yields. The Spearman rank correlation for the *trans* species is fairly high with both the ZN and FS methods, with the model accurately predicting low yields for a number of species. Supplementary Fig. 6 shows the ZN yield with the adiabatic model. The correlation among all species is reasonable, but for *trans* species is rather low. This is because of three molecules with zero predicted yield, all of which became trapped in the excited state. The diabatic model only predicts zero yield for one of these. Further, the diabatic model properly predicts zero yield for species **35**, while the adiabatic model does not. For these reasons the diabatic model has a fairly high correlation with experiment. Still, it is clear that excited state trapping of *trans* species is not a fully solved problem. Preferential sampling of excited state barriers may help to further address this issue in the future.

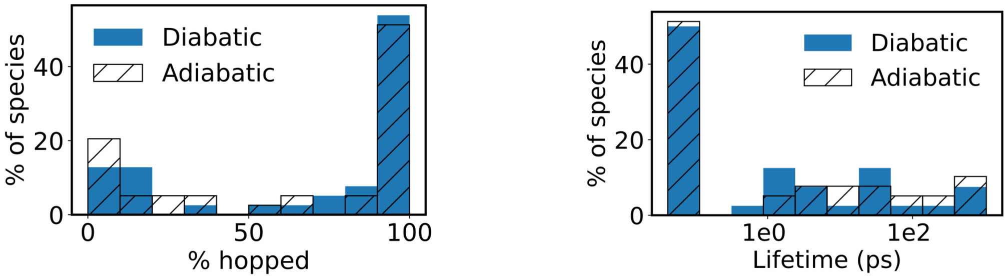

Supplementary Figure 4. Comparison of ZN hopping statistics in diabatic and adiabatic models. (a) Proportion of trajectories in each species that hopped to the ground state. (b) Excited state lifetime for each species.

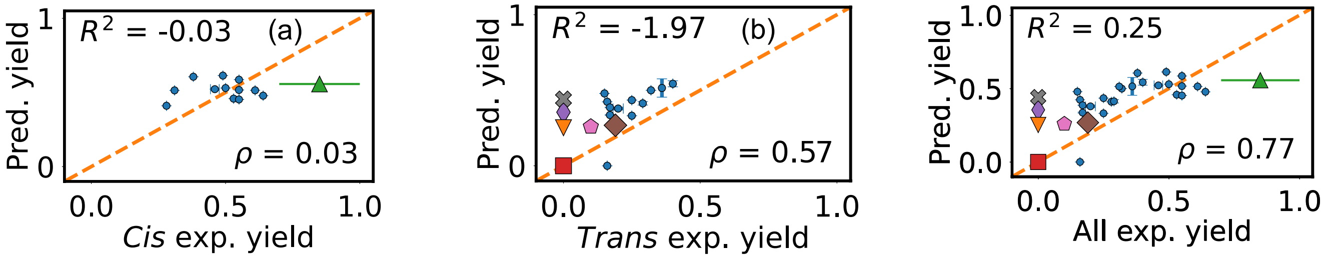

Supplementary Figure 5. Experimental vs. predicted ZN yields using the diabatic model. Prediction error bars represent one standard deviation of 1,000 bootstrapped samples. Experimental error bars represent reported uncertainties or the standard deviation from multiple observations, when applicable. (a) *Cis* isomers. (b) *Trans* isomers. (c) All species.

Supplementary Note 7. Architecture

All models were implemented in PyTorch [53]. The model hyperparameters are given in Supplementary Table IV, and an in-depth explanation of the PaiNN parameters can be found in Ref. [54]. Note that we used five convolutions instead of the three used originally, as this substantially improved model performance. Following DimeNet [55], we allowed the k values in the radial basis functions to be updated during training. For the adiabatic model, we predicted each property as a sum over per-convolution properties, which was also used in DimeNet. In particular, each convolution had a readout network to convert the atomic features to an output. The final property was obtained by summing each of these outputs.

Several variations on the architecture were tested. For example, we trained both two- and three-state diabatic models on 5,000 azobenzene configurations. We found that adding a third diabatic state significantly decreased the error for all properties. We then trained models using all possible three-state reference orderings and chose the best one. We also experimented with intensive pooling for off-diagonal energies (see Supplementary Note 15). In particular, we generated a molecular fingerprint through an attention-weighted average of atomic fingerprints. We then mapped this fingerprint to the d_{nm} for $n \neq m$. Even though the d_{nm} are intensive for $n \neq m$, this approach did not improve model predictions.

We also experimented with several adiabatic models. The model in the main text predicted E_0 and E_1 directly. This

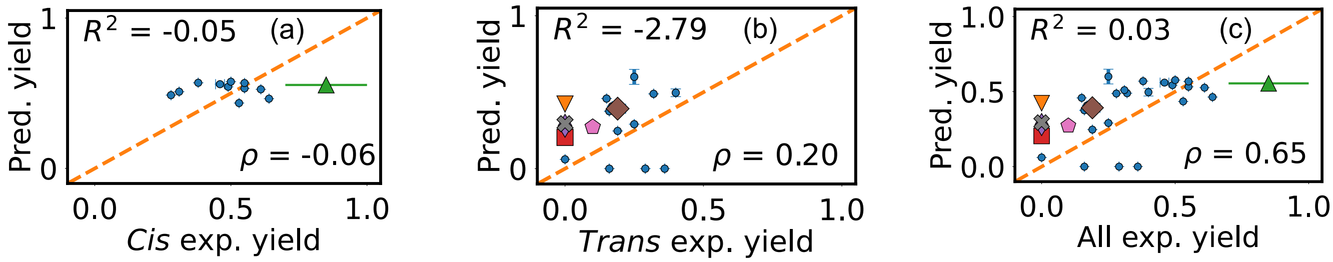

Supplementary Figure 6. Experimental vs. predicted ZN yields using the adiabatic model. Prediction error bars represent one standard deviation of 1,000 bootstrapped samples. Experimental error bars represent reported uncertainties or the standard deviation from multiple observations, when applicable. (a) *Cis* isomers. (b) *Trans* isomers. (c) All species.

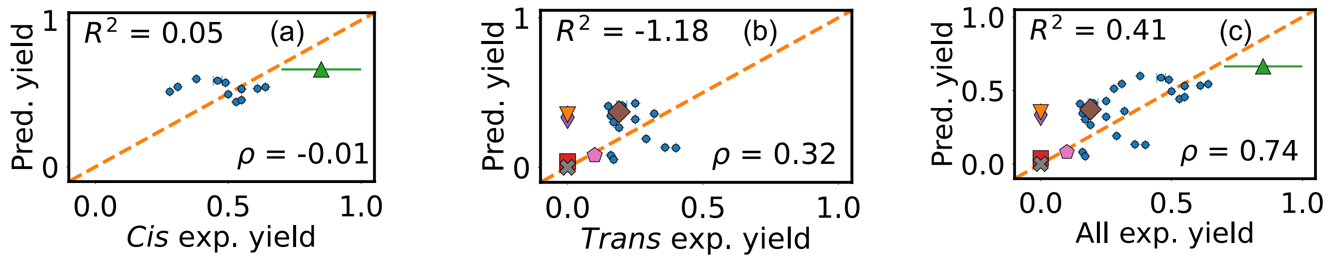

Supplementary Figure 7. Experimental vs. predicted FS yields using the diabatic model. Prediction error bars represent one standard deviation of 1,000 bootstrapped samples. Experimental error bars represent reported uncertainties or the standard deviation from multiple observations, when applicable.(a) *Cis* isomers. (b) *Trans* isomers. (c) All species.

approach was used in previous work for single-molecule non-adiabatic dynamics [6, 31, 56–60] and for the prediction of absorption spectra across chemical space [61]. We also examined three models that predicted E_0 directly and E_1 as the sum of E_0 and a learned gap. We tried three different pooling methods for the learned gap ΔE: taking the mean over atomwise gaps, taking an attention-weighted average over atomwise gaps, and applying a dense readout network to an attention-weighted sum of atomic fingerprints. All approaches performed quite poorly compared to learning E_0 and E_1 separately as summed atomwise energies. This is problematic, because only adiabatic models that predict E_1 as $E_0 + \Delta E$ can guarantee the positivity of the gap (e.g. by squaring ΔE or applying a softplus function). Hence the adiabatic model in the main text sometimes generated predictions with $E_1 < E_0$. This is impossible in the diabatic model by construction.

Supplementary Note 8. Training

The training set contained 562,037 geometries from 8,197 species. 5,000 geometries from 308 species were used for validation. The remaining 74,322 geometries from 40 species were held out for testing, so that the predicted yields of unseen species could be compared with experiment. A different random seed was used to determine the training and validation splits for each committee model, and also to initialize the different models. After training, we ran FS

ρ_{E_0}	ρ_{E_1}	$\rho_{\Delta E_{01}}$	ρ_{f_0}	ρ_{f_1}	ρ_{ref}	ρ_{nacv}
0.2	0.1	0.5	1	1	0.01	1

Supplementary Table V. Loss parameters used to train all diabatic models.

	lr	$\mathrm{lr}_{\mathrm{min}}$	$\rho_{\Delta E_{01}}$	ρ_{small}
Stage 1	10^{-4}	10^{-5}	0.5	0
Stage 2	10^{-5}	10^{-6}	1.0	100

Supplementary Table VI. Variable loss parameters used for training the adiabatic model. lr is the starting learning rate and $\mathrm{lr}_{\mathrm{min}}$ is the minimum learning rate, at which point training is stopped.

DANN-NAMD on 40 holdout species using the trained diabatic model. For each species we selected 50 geometries randomly and 50 by CI proximity (Supplementary Eq. (13)), for a total of 4,000 geometries. These geometries were used as the test set, giving the "unseen" statistics in Table I in the main text. The DANN-NAMD geometries from the diabatic network were used for all models, including the adiabatic and ablated ones in Supplementary Table I. The "seen" statistics were generated using the validation set. For all statistics, a phase correction was applied to $\vec{h}_{01}$ to minimize the prediction error, as in Supplementary Eq. (3).

The model for screening new azobenzene derivatives was trained on all species. Inclusion of the holdout species provided an additional 74,322 geometries. We used 631,367 geometries for training, 5,000 for validation, and 5,000 for testing. This corresponded to 8,215 training species, 332 validation species, and 303 test species.

Training was performed over energies and forces/force couplings in units of kcal/mol and kcal/mol/Å, respectively. Per-species reference energies were subtracted from each energy. These were obtained by summing atomic reference energies, computed using multi-variable linear regression from (atom type, count) to relaxed geometry energy. Configurations with 10-σ energy and force outliers were removed prior to training. Those with forces or energies $\geq$450 kcal/mol(/Å) from the mean were also removed. We found that more stringent removal of outliers led to less stable trajectories. For example, removing 3-σ outliers and maximal energies/forces of $\geq$300 kcal/mol(/Å) led to unstable ground state trajectories for six of the 40 unseen species. The 10-σ and 450 kcal/mol(/Å) criteria led to no diverging ground state trajectories. A small proportion of excited state trajectories were still unstable. We discarded all excited state trajectories that produced NaN geometries or energies, which were at most a few percent of the trajectories for a given species.

Models were trained with the Adam algorithm using a batch size of 60. Geometries were sampled for each batch using Supplementary Eq. (12), as described below. The loss was given by Eq. (6) in the main text, using parameters in Supplementary Table V. Note that the range of NACVs is approximately 10 times smaller than the range of forces. This means that $\rho_{\mathrm{nacv}} = 1$, $\rho_{f_i} = 1$ gives much higher weight to the forces. We experimented with a range of NACV coefficients between 0.1 and 10, and found that $\rho_{\mathrm{nacv}} = 1$ gave the best performance.

The learning rate was initialized to 10^{-4} and reduced by a factor of two if the validation loss had not improved in 10 epochs. The final model was selected as the one with the lowest validation loss. Training was performed on a single 32 GB Nvidia Volta V100 GPU, and took 13 days to complete.

For diabatic models, the training was stopped once the learning rate reached 10^{-5}. Adiabatic models were trained in a two-step process, using different loss functions in each stage. Each step ended once the learning rate fell below a certain value. The following loss function was used with different loss trade-offs at different stages:

$$\begin{aligned} \mathcal{L} &= \mathcal{L}_{\mathrm{core}} + \mathcal{L}_{\mathrm{small}}, \\ \mathcal{L}_{\mathrm{small}} &= \sum_{n>m} \rho_{\mathrm{small}} \cdot \mathrm{mse}\big(\Delta E_{nm}^{\mathrm{small}}\big), \end{aligned} \tag{4}$$

where $\mathcal{L}_{\mathrm{small}}$ penalizes errors in gaps under 0.2 eV. The parameters for each stage are given in Supplementary Table VI, and the ρ_E and ρ_f coefficients are the same as in Supplementary Table V. The first stage emphasized energy gaps and gradients, while the second stage emphasized small gaps to fine-tune the model near conical intersections. We also experimented with scheduled training and using $\mathcal{L}_{\mathrm{small}}$ for diabatic models, but did not find any improvements.

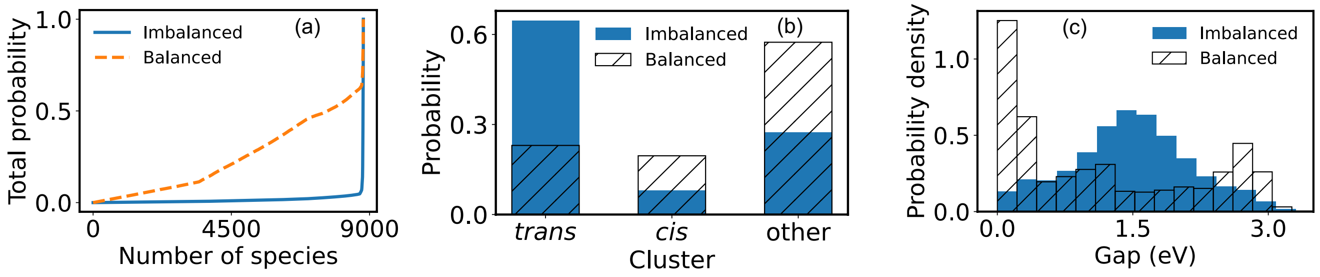

Supplementary Figure 8. Sampling probabilities for different geometries in the training set, with and without the custom sampler. (a) Cumulative probability $p(i)$ of sampling any species $\leq i$. The species are ordered from fewest to most geometries. (b) Probability of sampling a geometry in each of the three clusters. (c) Probability density of sampling a geometry with a given energy gap.

Supplementary Note 9. Balanced sampling

A custom data sampler was used during training because the dataset was imbalanced in the following ways. First, the combinatorial species only had a few geometries each, and so would be very rarely sampled during training. Second, there were more equilibrium *trans* geometries than *cis* geometries. Third, there were more equilibrium geometries in general than near-CI configurations. Our sampler addressed these imbalances by giving higher sampling probability to underrepresented species and/or configurations.

To explain the sampling procedure, let us define two types of sampling weights. Sampling weights that are balanced by species are denoted by w, and those that are not are denoted by v. For example, the weights $w_{\text{cluster}}(g_{i,A})$ and $v_{\text{cluster}}(g_{i,A})$ are both assigned to the ith geometry in species A, denoted $g_{i,A}$, based on the cluster of configurations that it belongs to. This cluster is denoted k, where $k \in$ (*cis*, *trans*, other). For a geometry $g_{i,A}$ in cluster k, the weights are given as follows:

$$
\begin{aligned}
w_{\text{cluster}}(g_{i,A}) &= \left(\frac{1}{n_{\text{spec}} \cdot n_{\text{clusters}}} \right) \left(\frac{1}{n_{k,A}} \right) \\
v_{\text{cluster}}(g_i, A) &= \left(\frac{1}{n_{\text{geoms}} \cdot n_{\text{clusters}}} \right) \left(\frac{n_A}{n_{k,A}} \right)
\end{aligned} \tag{5}
$$

Here $n_{k,A}$ is the number of geometries of species A in cluster k, and n_A is the number of geometries in species A. n_{spec} is the total number of species, n_{clusters} is the total number of clusters, and n_{geoms} is the total number of geometries. The above definitions ensure that there is an equal probability P_k of sampling any cluster k:

$$
\text{Using } w: \;\; P_k = \sum_A \sum_{g_{i,A} \in \; k} w_{\text{cluster}}(g_{i,A}) = \sum_A \frac{1}{n_{\text{spec}} \cdot n_{\text{clusters}}} = \frac{1}{n_{\text{clusters}}} \tag{6}
$$

$$
\text{Using } v: \;\; P_k = \sum_A \sum_{g_{i,A} \in \; k} v_{\text{cluster}}(g_{i,A}) = \sum_A \frac{n_A}{n_{\text{geoms}} \cdot n_{\text{clusters}}} = \frac{1}{n_{\text{clusters}}} \tag{7}
$$

Here we have used the fact that $\sum_{g_i \in \; k,A} 1 = n_{k,A}$, $\sum_A 1 = n_{\text{spec}}$, and $\sum_A n_A = n_{\text{geoms}}$. The difference between the

two weights is that w leads to balanced sampling among species, while v does not:

$$\text{Using } w: \; P_A = \sum_k \sum_{g_{i,A} \in k} w_{\text{cluster}}(g_{i,A}) = \sum_k \frac{1}{n_{\text{spec}} \cdot n_{\text{clusters}}} = \frac{1}{n_{\text{spec}}} \tag{8}$$

$$\text{Using } v: \; P_A = \sum_k \sum_{g_{i,A} \in k} w_{\text{cluster}}(g_{i,A}) = \sum_k \frac{n_A}{n_{\text{geoms}} \cdot n_{\text{clusters}}} = \frac{n_A}{n_{\text{geoms}}}. \tag{9}$$

Here we have used the fact that $\sum_k 1 = n_{\text{cluster}}$. We see that w gives equal weights for each species, whereas v gives weights proportional to the number of geometries in that species.

Similarly to the cluster weights, we define Zhu-Nakamura weights w_{ZN} and v_{ZN}, such that geometries with a higher hopping probability p_{ZN} are sampled more often. The corresponding expressions are:

$$w_{\text{ZN}}(g_{i,A}) = \left(\frac{1}{n_{\text{spec}} \cdot \sum_j p_{\text{ZN}}(g_{j,A})} \right) p_{\text{ZN}}(g_{i,A}) \tag{10}$$

$$v_{\text{ZN}}(g_{i,A}) = \left(\frac{1}{\sum_{j,B} p_{\text{ZN}}(g_{j,B})} \right) p_{\text{ZN}}(g_{i,A}). \tag{11}$$

The overall sampling weight for a given geometry is determined by each w and v, together with two user-defined weights: $P_{\text{spec}} \in [0,1]$, the importance of species balance, and $P_{\text{ZN}} \in [0,1]$, the importance of sampling geometries with high hopping rates. $P_{\text{cluster}} = 1 - P_{\text{ZN}}$ is the importance of sampling different clusters in a balanced way. The final sampling weight is then given by

$$\begin{aligned} p(g_{i,A}) = {} & P_{\text{ZN}} \left[P_{\text{spec}} \cdot w_{\text{ZN}}(g_{i,A}) + (1 - P_{\text{spec}}) \cdot v_{\text{ZN}}(g_{i,A}) \right] \\ & + P_{\text{cluster}} \left[P_{\text{spec}} \cdot w_{\text{cluster}}(g_{i,A}) + (1 - P_{\text{spec}}) \cdot v_{\text{cluster}}(g_{i,A}) \right]. \end{aligned} \tag{12}$$

In applying Supplementary Eq. (12) we used $P_{\text{spec}} = 0.6$ and $P_{\text{cluster}} = 0.5$. We defined a geometry as *cis* or *trans* if its RMSD with respect to the corresponding reference structure was ≤ 0.25 Å, and "other" otherwise. In contrast to diabatic reference geometries, the RMSD was computed using all atoms, not just the central CNNC atoms. Note that while our clustering approach was specific to *cis-trans* isomerization, many black-box clustering methods exist, such as hierarchical clustering [62]. Any of these methods could have been used in place of our user-defined clusters.

In principle p_{ZN} should depend on trajectory-specific factors such as velocity, and is therefore not a function of geometry alone. However, during simulations, hops almost always occurred below 0.5 eV. Further, since p_{ZN} is approximately exponential in the square of the gap, we approximated it with

$$p_{\text{ZN}}(\Delta E) \approx \exp(-\Delta E^2 / (2 \Delta E_0^2)), \tag{13}$$

where ΔE is the energy gap and $\Delta E_0 = 0.15$ eV. This choice of ΔE_0 gave $p(\Delta E = 0.5 \text{ eV}) \approx 3 \times 10^{-3}$. This meant that geometries were usually selected only if their gap was under 0.5 eV.

The sampling probabilities for the training set are shown in Supplementary Fig. 8. Panel (a) shows the cumulative probability of sampling different species, with the compounds ordered from fewest to most geometries. Without the custom sampler the probability was quite small for most molecules. This was because the majority were combinatorially generated and had few geometries. 90% of the probability was contained in the last 41 species. This probability was reduced to 35% when using the balanced sampler. Panel (b) shows that most geometries were *trans* isomers and few were *cis*. The custom sampler gave approximately equal probability to the two isomers, and the highest probability to the *other* group. Most geometries in this group had small gaps, and hence were highly weighted by w_{ZN} and v_{ZN}. This is demonstrated in panel (c), which shows that near-CI geometries had the highest probabilities when using the balanced sampler.

Supplementary Note 10. Dynamics

Simulations were performed in two stages. First the starting geometries and velocities were generated for each NAMD trajectory. For *ab initio* simulations we optimized the ground state geometry of each species and computed

its normal modes. These modes were used to sample geometries and velocities from the harmonic oscillator Wigner distribution at temperature $T = 300$ K [63]. For neural trajectories we ran classical MD for 15 ps with the Nosé-Hoover thermostat [64, 65], and selected random samples to start the NAMD trajectories. Classical MD was implemented with ASE [66]. The effective mass Q was set to $(3N-6)\cdot\tau^2 k_{\mathrm{B}}T$, where N is the number of atoms, k_{B} is Boltzmann's constant, $T = 300$ K, and $\tau = 25$ fs is the relaxation time. Initial atomic velocities were sampled from a Maxwell-Boltzmann distribution at 300 K. Optimized geometries were used for the initial atomic positions. The total linear and angular momenta of the system were set to zero, and the target kinetic energy was set to $(3N-6)\cdot k_{\mathrm{B}}T/2$. The time step of the simulation was set to 0.5 fs, and the neighbor list of the system was updated every 10 time steps (5 fs). All pairs of atoms within with 7 Å were considered neighbors. In each step the distance between all pairs of neighbors was computed, and only pairs within 5 Å of each other were used in the model. This procedure meant that atoms entering the 5 Å cutoff between neighbor list updates would not be missed (i.e., an extra 2 Å "skin" was addded). All ground state and excited state trajectories in this work were propagated with the velocity Verlet algorithm [67]. Frames for NAMD were taken only after the first 1 ps of ground state MD to allow for equilibration.

Next we ran ZN dynamics [7, 28, 68] using N_{trj} trajectories, where N_{trj} was 10 during the active learning cycle and 500 during final inference. Hops were restricted to gaps ≤ 0.5 eV to avoid unphysical transitions, the time step was set to 0.5 fs [7, 28, 68], and the neighbor list was again updated every 10 steps. Excited state trajectories were run for 1.5 ps during active learning and 5 ps for final inference. All other details of the implementation can be found in Refs. [7, 28, 68]. For each species we performed inference in parallel over 100-250 geometries at a time, depending on the number of atoms in the molecule. This was done by batching together geometries from 100-250 trajectories and evaluating the model on the batch. The batch size depended on the size of the molecule, which determined the GPU memory consumption. FS simulations were also performed for final inference. The ZN parameters above were used for FS DANN-NAMD of molecules in the holdout set. For virtual screening we used 100 trajectories and 2 ps of excited state dynamics. Species with a hopping rate under 10%, or with molecular graphs that changed during ground state dynamics, were deemed unreliable. These molecules were excluded from Fig. 4 in the main text, but not from the average hopping percentage reported in the main text.

The quantum yield was calculated as $Y = n_R/n_T$, where n_R is the number of reactive trajectories and n_T is the total number of trajectories. The uncertainty was computed as the standard deviation of 1,000 bootstrapped samples. To identify reactivity, we computed the RMSD between the CNNC atoms in the final frame and the corresponding atoms in the optimized *cis*/*trans* geometries. A trajectory was considered reactive if it started as *cis* and ended closer to *trans*, or vice-versa. Trajectories that ended in the excited state were excluded from n_R and n_T. The yield was reported as 0 ± 0 if all trajectories ended in the excited state.

Supplementary Note 11. Fewest switches implementation

Tully's surface hopping propagates the nuclei on one electronic surface at a time, called the active surface. The expansion coefficients of the electronic wavefunction are also propagated, and are used to make stochastic changes in the active surface. The coefficient vector $\mathbf{c}$, expressed in a basis denoted by "rep", evolves as [69]

$$\frac{d}{dt}\mathbf{c}^{\mathrm{rep}} = -\frac{i}{\hbar}[\mathbf{H}^{\mathrm{rep}} - i\mathbf{T}^{\mathrm{rep}}]\mathbf{c}^{\mathrm{rep}}. \tag{14}$$

The Hamiltonian matrix is $\mathbf{H}^{\mathrm{rep}} = \langle\psi_n^{\mathrm{rep}}|\, H\, |\psi_m^{\mathrm{rep}}\rangle$, and the coupling term is

$$(\mathbf{T}^{\mathrm{rep}})_{nm} = \left\langle \psi_n^{\mathrm{rep}}(\vec{R}(t)) \middle| \frac{\partial}{\partial t} \middle| \psi_m^{\mathrm{rep}}(\vec{R}(t)) \right\rangle = \vec{v}\cdot\vec{k}_{nm}^{\mathrm{rep}}, \tag{15}$$

where $\vec{v}$ is the classical velocity of the nuclei. In principle the nuclei can be propagated on a PES in any electronic basis, and hops can be performed between states in that basis. For example, nuclei could be propagated on diabatic PESs and hops could occur between diabatic states. However, it is well-established that surface hopping in the adiabatic basis gives the best results [19]. Hence the nuclei should be propagated in the adiabatic basis, and hops should be decided using $\mathbf{c}^{\mathrm{ad}}$.

While the adiabatic basis should be used for hopping, $\mathbf{c}$ can be propagated in the diabatic basis, and then transformed into the adiabatic basis for all subsequent manipulations (e.g. deciding on hops, adding decoherence, etc.). Indeed, using Supplementary Eq. (14) in the adiabatic basis can require small time steps for so-called trivial crossings, where derivative NACVs are quite narrow and large [70]. Since global diabatic states are almost never available in *ab initio* simulations, a local diabatization method is often used to propagate $\mathbf{c}$ [3, 70]. This method is very stable and can be used with a fairly large time step. In this work we have a global diabatic basis that can replace local diabatization. Following Refs. [69, 70], we then propagate $\mathbf{c}$ as follows:

$$\mathbf{c}^{\rm ad}(t+\Delta t) = \mathbf{P}^{\rm ad}(t, t+\Delta t)\,\mathbf{c}^{\rm ad}(t), \tag{16}$$

$$\mathbf{P}^{\rm ad}(t, t+\Delta t) = \mathbf{U}^{\dagger}(t+\Delta t)\,\mathbf{P}^{\rm d}(t, t+\Delta t)\,\mathbf{U}(t) \tag{17}$$

$$\mathbf{P}^{\rm d}(t, t+\Delta t) = \Pi_{k=1}^{K}\, \exp\left[-i\mathbf{H}^{\rm d}_{k}\Delta t/K\right] \tag{18}$$

$$\mathbf{H}^{\rm d}_{k} = \mathbf{H}^{\rm d}(t) + \frac{k}{K}\left[\mathbf{H}^{\rm d}(t+\Delta t) - \mathbf{H}^{\rm d}(t)\right]. \tag{19}$$

Here $\mathbf{P}^{\rm ad}(t, t+\Delta t)$ is the propagator of $\mathbf{c}^{\rm ad}$ from t to $t+\Delta t$ (Supplementary Eq. (16)), and Δt is the timestep. The adiabatic propagator is a transformation of the diabatic propagator into the adiabatic basis (Supplementary Eq. (17)), using the transformation matrix $\mathbf{U}$ (Eq. (2) in the main text). The diabatic propagator is a matrix product of K sub-propagators (Supplementary Eq. (18)), where K is the number of electronic substeps for each nuclear step. "exp" denotes a matrix exponential, not element-wise exponentiation. The $k^{\rm th}$ sub-propagator uses a linear interpolation between $\mathbf{H}^{d}(t)$ and $\mathbf{H}^{d}(t+\Delta t)$ to approximate $\mathbf{H}_{d}(t+k\Delta t/K)$ (Supplementary Eq. (19)). The diabatic Hamiltonian is produced by the neural network model. The probability of hopping from state n to m is [69]

$$p_{n\to m} = \left(1 - \frac{|c^{\rm ad}_{n}(t+\Delta t)|^2}{|c^{\rm ad}_{n}(t)|^2}\right) \frac{\mathrm{Re}\left[c^{\rm ad}_{m}(t+\Delta t)\left(P^{\rm ad}_{mn}\right)^{*}\left(c^{\rm ad}_{n}(t)\right)^{*}\right]}{|c^{\rm ad}_{n}(t)|^2 - \mathrm{Re}\left[c^{\rm ad}_{n}(t+\Delta t)\left(P^{\rm ad}_{nn}\right)^{*}\left(c^{\rm ad}_{n}(t)\right)^{*}\right]}, \tag{20}$$

where $\mathrm{Re}(x)$ is the real part of x. Note that while our approach follows that of SHARC [69], we have written our own code and made it publicly available [71]. Our repository also contains code for surface-hopping with the ZN method.

In this work we initialized $\mathbf{c}^{\rm ad}(t=0) = [0, 1, 0]$, used Supplementary Eqs. (16)-(19) to generate $\mathbf{c}^{\rm ad}(t+\Delta t)$, and computed the hopping probability with Supplementary Eq. (20). $\mathbf{c}^{\rm ad}$ was expressed in a three-state basis because our model used three diabatic states, and hence $\mathbf{U}$ had three dimensions. Since the model was only trained on the first and second adiabatic energies, we set $p = 0$ for all transitions to the third state. Hops to state m were performed if [69]

$$\sum_{i=1}^{m-1} p_{n\to i} < r \leq \sum_{i=1}^{m-1} p_{n\to i} + p_{n\to m}, \tag{21}$$

where $0 \leq r \leq 1$ is a random number. The momentum was rescaled after each hop to conserve the total energy. The momentum was multiplied by a constant factor, rather than being re-scaled in the direction of the NACV, to avoid the overhead of a NACV calculation (see below) [3]. If the factor was complex—a so-called "frustrated" hop—then no transition was made [3].

We also tested propagation in the adiabatic basis. In this case we used the NACV to evaluate $\mathbf{T}$, and constructed $P^{\rm ad}$ from the interpolation of $\mathbf{H}^{\rm ad} - i\mathbf{T}$ [69]. Both Supplementary Eq. (20) and Tully's original hopping expression [2] were used. The momentum was re-scaled in the direction of the NACV [49]. In all cases the results were very similar to those of the diabatic basis. Diabatic propagation was ultimately chosen because of its reported stability [70], and because of its computational efficiency. In particular, the diabatic propagation requires only diabatic energies and one adiabatic gradient. It therefore uses only one forward and one backward pass through the neural network. By contrast, constructing the NACVs requires gradients for each diabatic element (Eq. (1) in the main text). For three diabatic states, this corresponds to one forward pass and six backward passes. While shared convolution layers and caching mean that N gradients do not take $N\times$ as long as one gradient, we found that they still added significant time. Hence diabatic propagation was chosen as the most efficient method.

The decoherence correction of Ref. [72] was used to counter the over-coherence of Tully's original method. A sign correction was also used to remove random sign changes in the eigenvectors of $\mathbf{H}_d$. The sign correction A_m for the mth eigenvector $\mathbf{v}_m$ was computed as

$$A_m = \text{sgn}(S_{n'm}) \tag{22}$$

$$S_{nm} = [\mathbf{U}^\dagger(t)\mathbf{U}(t+\Delta t)]_{nm} \tag{23}$$

$$n' = \text{argmax}_n\{|S_{nm}|\}. \tag{24}$$

The transformation matrix and NACVs were corrected through

$$U_{nm}(t+\Delta t) \to A_m U_{nm}(t+\Delta t) \tag{25}$$

$$\vec{k}_{nm}(t+\Delta t) \to A_n A_m \vec{k}_{nm}(t+\Delta t) \tag{26}$$

$$\vec{h}_{nm}(t+\Delta t) \to A_n A_m \vec{h}_{nm}(t+\Delta t). \tag{27}$$

Note also that $S_{nm} \approx \langle \psi_n^{\text{ad}}(t) | \psi_m^{\text{ad}}(t+\Delta t) \rangle$:

$$\begin{aligned} \langle \psi_n^{\text{ad}}(t) | \psi_m^{\text{ad}}(t+\Delta t) \rangle &= \sum_{ij} U_{in}^*(t) U_{jm}(t+\Delta t) \langle \psi_i^{\text{d}}(t) | \psi_j^{\text{d}}(t+\Delta t) \rangle \\ &\approx \sum_{ij} U_{ni}^\dagger(t) U_{jm}(t+\Delta t) \langle \psi_i^{\text{d}}(t) | [(1 - (\vec{v}\Delta t) \cdot \nabla_R) | \psi_j^{\text{d}}(t) \rangle] \\ &= \sum_{ij} U_{ni}^\dagger(t) U_{jm}(t+\Delta t) \langle \psi_n^{\text{d}}(t) | \psi_m^{\text{d}}(t) \rangle \\ &= [\mathbf{U}^\dagger(t)\mathbf{U}(t+\Delta t)]_{nm} = S_{nm}. \end{aligned} \tag{28}$$

Here we used the fact that there is no derivative coupling between diabatic states, and that the diabatic states at a given time are orthonormal (Supplementary Eq. (35)).

All simulations were performed with a time step of 0.5 fs and $K = 25$ substeps for electronic propagation. Unlike in ZN dynamics, hops were not restricted to gaps under 0.5 eV. We found that restricting hops improved the ZN results but hurt the FS results. For example, with no maximum gap, most neural ZN trajectories starting from *trans*-azobenzene hopped at ~ 1.5 eV. This did not match the results of Ref. [28], which used *ab initio* ZN with the same level of DFT theory. After adding the restriction, most hops occurred at gaps under 0.1 eV, in agreement with Ref. [28]. By contrast, most FS hops occurred under 0.1 eV even without a maximum restriction, though some still occurred at large gaps. The lifetime and yield without a maximum also better matched previous calculations.

Supplementary Note 12. Active learning

New geometries were selected for QC calculations using two different criteria. In the first three active learning loops, new geometries were chosen based on the prediction variance of two neural networks. Our aim was to select geometries with fairly uncertain predictions. We did not want geometries with extremely high uncertainty, as these usually corresponded to broken graphs that were outside the target learning space for the model. We therefore used a log-normal target distribution for the uncertainty. Target uncertainties were randomly sampled from this distribution, and geometries with the closest variance to the targets were selected. The log-normal probability of obtaining a sample x is given by $P(x) \propto \exp\left[-(\ln(x/s) - \mu)^2/2\sigma^2\right]/(x\sigma\sqrt{2\pi})$, where s, σ and μ are positive numbers. Under this distribution, both completely certain and completely uncertain predictions have zero probability, with a peak for predictions with medium uncertainty. The decay of the probability at large uncertainty is slow, such that highly uncertain geometries can still be selected with reasonable probability. We set $\mu = 0$, $\sigma = 1$, and $s = 7$, giving a target distribution with a mode of 2.5 kcal/mol(/Å) and a mean of 11.5 kcal/mol(/Å). Committee variances were computed for both forces and energies for each electronic state, and the largest variance was compared with the target variance from the distribution. For each species we selected 16 geometries from ground state MD and 33 from surface hopping.

Only geometries from the 164 literature species were chosen. This led to approximately 8,000 new data points in each active learning cycle. Other, simpler methods of geometry selection are certainly possible; for example, random selection is also known to work quite well [73, 74]. Our approach successfully increased the model quality throughout active learning, but we did not thoroughly compare it to other methods. Such a comparison may be of interest in the future.

In the next two loops we used only azobenzene, with the goal of densely sampling the CI region. Half the geometries were selected randomly from the excited state dynamics, and half were selected based on the gap. Because the model overestimated the gap in uncertain regions, we did not want to simply select geometries with low predicted gap. Rather, in each trajectory we identified avoided crossing geometries, and assigned equal sampling probability to all geometries before and after that crossing, up to a maximum predicted gap of 1.7 eV. An avoided crossing geometry was defined as having a gap that was lower than in the previous and subsequent time step. In this way we aimed to identify regions that could have small gaps, even if the model did not predict them to be so.

Supplementary Note 13. Validation

A. *Ab initio NAMD*

Ab initio simulations were used for unsubstituted *cis* and *trans* azobenzene. Starting configurations and velocities were generated with *ab initio* MD using Q-Chem to drive the dynamics. Ten MD simulations were performed for each species. Each simulation was initiated with a different geometry produced by ground-state neural network MD. The simulations were run for 10 ps each using a time step of 0.5 fs, with the the BP86 functional [75, 76] and 6-31G* basis [25]. Initial velocities were sampled from a thermal distribution at 300 K, with rotation and translation projected out. The nuclei were propagated with the Velocity Verlet algorithm. A Nosé-Hoover chain of length 5, timescale $\tau = 25$ fs, and temperature $T = 300$ K was used as a thermostat. The Fock extrapolation order was set to six, and twelve Fock matrices from previous steps were used in the extrapolation.

167 and 178 excited state trajectories were generated for *trans* and *cis* azobenzene, respectively. Each trajectory was initialized with a different set of coordinates and velocities, randomly sampled from the ten ground state simulations after 1 ps of equilibration. Spin-flip TDDFT was used with the BHHLYP functional [24] and 6-31G* basis. DIIS with geometric direct minimization (GDM) [77] was used to improve SCF convergence. We found this to be critical: with the usual DIIS algorithm, the SCF cycle failed to converge for 36% of trajectories. This usually occurred within the first 200 fs.

The dynamics were run with in-house scripts, which can be found at `https://github.com/learningmatter-mit/NeuralForceField`. We propagated the elements of the electronic wavefunction in the adiabatic basis, and corrected the sign of the force NACV by minimizing its change from the previous step. The momentum was re-scaled in the direction of the NACV after a hop [49]. All other parameters were unchanged from the DANN-NAMD simulations. *Cis* trajectories were propagated for 200 fs, and *trans* trajectories for a maximum of 1.5 ps. For the former, we extended trajectories that had hopped within the last 50 fs, or not hopped at all, until at least 50 fs had passed since they hopped. For the latter, we stopped a trajectory if it had lasted at least 500 fs and had hopped more than 300 fs earlier.

B. *Transfer learning*

To validate the yield results for substituted compounds, we performed DANN-NAMD for the top candidates using a set of highly accurate models. Each model was fine-tuned for a single species only, which allowed it to achieve high accuracy on that one molecule. This transfer learning strategy was used in place of *ab initio* NAMD because the latter would be prohibitively slow for all but the smallest molecules. For example, consider molecule **169**, which has only 54 atoms. We found that a single gradient or NACV calculation for this species took approximately 50 minutes with 8 CPU cores. Since *trans* derivatives must be simulated for at least 1 ps, and since the time step must be no larger than 0.5 fs, we would need to perform 2,000 QC calculations for each trajectory. Assuming parallel calculation of the

lr	lr_{min}	patience	factor	batch size	ρ_{E_0}	ρ_{E_1}	$\rho_{\Delta E_{01}}$	ρ_{f_0}	ρ_{f_1}	ρ_{ref}	ρ_{nacv}
10^{-4}	10^{-5}	50	0.5	1	0.3	0.3	0.5	1	1	0	1

Supplementary Table VII. Training parameters used for transfer learning. "Patience" refers to the number of epochs without an improvement in validation loss before the learning rate is reduced. "Factor" is the amount by which the learning rate is lowered.

NACVs and gradients at each step, an *ab initio* simulation would take 70 days. By contrast, the fine-tuning approach allows us to perform highly accurate simulations for tens to hundreds of species.

Each new model was refined from the original network using QC data from a single species. The initial training geometries were sampled from DANN-NAMD simulations with the original DANN model. A committee of three DANN models, each trained on the entire dataset, was used to select the initial geometries (see below). Three fine-tuned models were then trained. Each was initialized with a different random seed and trained with different data splits. 90% of the data was used for training and 10% for validation. The first model was subsequently used for DANN-NAMD. Geometries were selected from these simulations using the newly-trained committee, and each geometry received QC calculations. This data was added to the training set, each committee model was re-trained, and the cycle was repeated as in Fig. 2(b) in the main text. After each cycle we evaluated the model accuracy using the geometries generated by DANN-NAMD.

Initially we selected only 50 geometries in each round of active learning. Once we narrowed our focus to two species, we sampled 500 geometries in each round. The geometries were sampled according to the following four strategies. One third was sampled randomly. One third was chosen by the prediction variance in the excited state forces. Those with the highest variance were selected. We used only the uncertainty in the forces, and not the energies, because the former is a better indicator of trajectory instability [62]. One sixth was chosen by proximity to a CI (Supplementary Eq. (13) with $\Delta E = 0.2$ eV). The final sixth was chosen to sample excited-state barriers. In particular, we sampled geometries with probability

$$p \propto \exp\left[(E - E_{\mathrm{min}})/(k_{\mathrm{B}}T)\right]. \tag{29}$$

Here E is the excited state energy, E_{min} is the minimum excited sate energy in the trajectory, and $T = 300$ K. We only sampled configurations from 50 fs or later in the simulations. This was done to avoid the initial high-energy geometries encountered before relaxation. Supplementary Eq. (29) is inversely proportional to the room-temperature Boltzmann probability, and thus assigns the highest weight to the highest-energy configurations.

Transfer learning has previously been used to account for solvent effects [74] and to reach higher levels of QC theory [74, 78] with only a small portion of the training set. Typically the majority of the network weights are frozen during re-training. This leaves modifiable parameters in only the final few layers. However, we found that the best results were obtained without any parameter freezing. We therefore allowed all parameters to be modified during re-training. Further, we found it best to start with the normal learning rate rather than a reduced one. We also did not use a reference loss, as its effect on the original DANN results was minor, and possibly even harmful. The full set of training parameters can be found in Supplementary Table VII. All trajectory parameters were unchanged from the screening phase. For final inference we performed DANN-NAMD for 5 ps with 2,000 trajectories, using 500 ps of ground state MD.

The accuracy of the fine-tuned networks is shown in Supplementary Tables VIII and IX. Statistics are shown for 500 geometries that were sampled from DANN-NAMD for each molecule using the final trained networks. The model errors are well below 1 kcal/mol(/Å) for geometries sampled by all methods other than uncertainty. The error on the uncertainty-selected configurations is under 2 kcal/mol(/Å) in all cases. Since these geometries are specifically chosen to have the highest error, and since their errors are still rather small, we can be confident in the accuracy of the models. We note, however, that the average error for the uncertain geometries depends on how many trajectories are run. Running more trajectories means sampling more configurations, and hence finding more geometries with high uncertainty. As mentioned above, we ran 100 trajectories for 5 ps each for both screening and transfer learning (2,000 trajectories were used for the final predictions). We saved frames every 15 fs, leading to 33,333 geometries in total. Since we picked the 167 most uncertain frames, our sample is roughly the same as choosing the top 0.5% of all geometries with the highest error.

Sampled by	Metric	E_0	E_1	ΔE_{01}	$\vec{F}_0$	$\vec{F}_1$	$\vec{h}_{01}$
ZN	MAE (↓)	0.48	0.42	0.44	0.83	0.82	0.52
	R^2 (↑)	1.00	1.00	0.96	0.99	0.99	0.86
Barrier	MAE (↓)	0.62	0.93	0.86	0.67	0.82	0.66
	R^2 (↑)	0.99	0.99	0.99	1.00	0.99	0.88
Random	MAE (↓)	0.74	0.80	0.86	0.71	0.77	0.89
	R^2 (↑)	0.99	0.99	0.98	1.00	1.00	0.88
Uncertainty	MAE (↓)	0.74	1.26	1.09	0.83	1.36	1.04
	R^2 (↑)	0.99	0.99	0.99	0.99	0.97	0.73

Supplementary Table VIII. Test set statistics for the final TL model of species **165**. Results are divided by the method used to select samples. "ZN" uses Zhu-Nakamura gap-based sampling [Supplementary Eq. (13)] and "barrier" uses Supplementary Eq. (29). 1,244 geometries were used for fine-tuning.

Sampled by	Metric	E_0	E_1	ΔE_{01}	$\vec{F}_0$	$\vec{F}_1$	$\vec{h}_{01}$
ZN	MAE (↓)	0.67	0.54	0.39	0.87	0.84	0.64
	R^2 (↑)	0.98	0.98	0.99	0.99	0.99	0.82
Barrier	MAE (↓)	0.89	0.74	0.41	0.61	0.57	0.51
	R^2 (↑)	0.99	0.95	1.00	1.00	1.00	0.98
Random	MAE (↓)	0.58	0.66	0.51	0.67	0.70	0.78
	R^2 (↑)	0.99	1.00	1.00	1.00	1.00	0.96
Uncertainty	MAE (↓)	0.72	1.66	1.90	0.83	1.53	1.01
	R^2 (↑)	0.99	0.93	0.97	0.99	0.95	0.92

Supplementary Table IX. As in Supplementary Table VIII, but for species **169**. 2,445 geometries were used for fine-tuning.

One reason that we did not use transfer learning for the unsubstituted compounds was severe spin contamination. This is an artifact of unrestricted SF-TDDFT, and is a well-documented problem in NAMD for *trans* azobenzene [28]. After the first round of active learning, we found that the force error for the unsubstituted models increased to 8 kcal/mol/Å. This error did not drop significantly with more data, even though the new geometries were fairly close to the Franck-Condon region. This issue did not occur with any of the derivatives. We found that the error was correlated with $\langle S^2 \rangle$, and reasoned that our method of singlet selection (Supplementary Methods D) was likely choosing triplet excited states. This highlights the issues inherent in SF-TDDFT, and reinforces the need for low-cost, spin-complete alternatives [79–81].

Lastly, we note that both the *ab initio* and transfer-learned *cis* quantum yields were noticeably higher than in other SF-TDDFT studies [28]. This is likely because we used MD to initiate the trajectories, rather than normal-mode or Wigner sampling based on the harmonic approximation. Our experiments showed that normal-mode sampling led to decreased *cis* yields, closer to those reported in Ref. [28]. Unlike the *trans* isomer, *cis* azobenzene is somewhat flexible, with significant torsions occurring during ground-state MD. This indicates that the harmonic approximation should be avoided when possible. Indeed, using MD ground state sampling together with FS dynamics for *cis* azobenzene, we obtained a yield of 60 ± 4%; this is in excellent agreement with experimental values in non-polar solution, which are close to 55% on average [44]. It is in much better agreement than the value of 34% obtained with FS surface hopping in Ref. [28]. Since we used the same electronic structure method and the same surface hopping approach, we can be confident that the difference is mainly due to MD sampling.

C. *Conical intersection pathways*

We labeled the CI pathway of each *trans* trajectory according to its last hopping geometry. Each geometry was compared to two reference planar CIs and two reference rotational CIs. The trajectory was labeled by the reference CI that was the closest to the hopping geometry. That is, if a trajectory hopped at a geometry closer to one of the

rotational CIs, it was labeled a rotational trajectory, and similarly for the planar CI.

The reference CIs were chosen from the set of all hopping geometries in the trajectories. The two rotational CIs were those with dihedral angles closest to 90° and 270°, respectively, and $\max(\alpha_{\text{CNN}}, \alpha_{\text{NNC}})$ closest to 138°. The two planar CIs were those with dihedral angles closest to 186° and 174°, respectively, and both $\max(\alpha_{\text{CNN}}$ and $\alpha_{\text{NNC}})$ closest to 148°. The angles are those of the optimized CI geometries in Ref. [48]. For the derivative **169**, we further optimized each of the reference structures to minimize the gap and hence obtain a true CI. For each trajectory we computed the RMSD between the CNNC atoms of the hopping geometry and the CNNC atoms of the reference CIs.

We additionally enforced that a hopping geometry could only be considered planar if $|180 - \theta| \leq \theta_0$, where θ is the CNNC dihedral angle and θ_0 is a cutoff value. We chose $\theta_0 = 75°$ for azobenzene and 65° for **169**. Without this constraint, many of the hopping geometries labeled "planar" in **169** actually led to isomerization. This made a dihedral constraint necessary. On the other hand, when we only labeled all geometries with $|\theta - 180| \leq 45°$ as planar [48], we found through visual inspection that many non-reactive CI geometries were mislabeled as rotational. Using the minimal distance to a reference CI, together with a modest dihedral constraint, led to the most qualitatively reasonable results. We confirmed that none of the geometries labeled as "planar CI" with our metric led to switching, which further reinforced the soundness of our approach.

Supplementary Note 14. Figure details

A. *Computational speed-up*

Here we describe how the speeds of ML and QC calculations were computed. For QC, we first computed the run time of one gradient calculation on a single geometry, denoted t_{calc}. The node time was then calculated as $t_{\text{node}} = t_{\text{calc}}/n_{\text{calc}}$, with $n_{\text{calc}} = (\text{cores per node})/(\text{cores per job})$. We assumed 40 cores per node. All QC jobs were performed with 8 cores, and so n_{calc} was equal to 5.

For ML we performed a batched calculation on ten copies of each geometry, and computed one gradient. The node time was computed as $t_{\text{node}} = t_{\text{calc}}/n_{\text{calc}}$, with $n_{\text{calc}} = 10 \cdot (\text{total memory per gpu})/(\text{memory used in calculation}) \cdot (\text{GPUs per node})$. We set (total memory per gpu) = 32 GB and (GPUs per node) = 2. We used a script that performed one batched calculation on ten copies of a random geometry, and re-ran it 7,000 times. For each iteration we used the `nvidia-smi` command with the PID of the current job to access the GPU memory. We re-ran the script many times, rather than running many calculations in one script, because `nvidia-smi` does not account for all freed memory until a job is finished. That is, after one calculation is finished, `nvidia-smi` shows that GPU memory is still being occupied by the job. This occurs even after all local variables are deleted. Hence running multiple calculations in one script would yield an overestimated GPU memory.

In the above calculation, we implicitly assumed that multiplying the batch size by x would lead to an x-fold increase in memory. In practice we have found that the memory is increased by less than that. This means that the ML speedup in Fig. 3 in the main text is a conservative estimate.

B. *Diabatic energies*

Since three diabatic states were used in the DANN model, and since all three were coupled near the CI, it would be incorrect to plot only two states in Fig. 3(b) in the main text. We therefore applied a fixed rotation matrix, $\mathbf{U} \neq \mathbf{U}(\vec{R})$, to generate a new diabatic Hamiltonian with only two coupled states. The new diabatic Hamiltonian was given by $\mathbf{H}'_d(\vec{R}) = \mathbf{U}^\dagger \mathbf{H}_d(\vec{R})\mathbf{U}$. Note that any position-independent rotation matrix can be brought outside the gradient in Eq. (1) in the main text, and so $\mathbf{H}'_d$ is still diabatic. The rotation matrix was chosen to diagonalize $\mathbf{H}_d$ at the CI, so that $\mathbf{U}^\dagger \mathbf{H}_d(\vec{R}_{\text{CI}})\,\mathbf{U} = \text{diag}(\{E\})$. This led to $d'_{00} = d'_{11}$ and $d'_{01} = 0$ at the CI. Hence the lowest two rotated states were the most important contributors to E_0 and E_1, and were therefore used in Fig. 3(b) in the main text.

Supplementary Note 15. Intensive and extensive quantities

The DANN model predicts each property by summing over atomic contributions, just as in the PaiNN model. This guarantees size-extensivity. However, as explained below, the off-diagonal elements of $\mathbf{H}_d$ should be intensive, in the sense that atoms not involved in the excitation should not contribute to these values. Nonetheless, we found that summation for these terms gave better results than averaging. This was true even when using a learnable weighted average. Atom-wise summation can still generate accurate d_{nm}, because the readout network can simply map unimportant atoms to zero.

To demonstrate extensivity and intensivity, consider two uncoupled subsystems, A and B. The total clamped nucleus Hamiltonian is $H(\vec{r}, \vec{R}) = H^A(\vec{r}, \vec{R}) + H^B(\vec{r}, \vec{R})$. Let the excitation of interest be in subsystem A. In this case the adiabatic states involve only excitations in subsystem A, so that the nth diabatic wave function is written as

$$\psi_{\text{d},n}(\vec{r}; \vec{R}) = \psi^B_{\text{ad},0}(\vec{r}; \vec{R}) \sum_k U_{nk} \psi^A_{\text{ad},k}(\vec{r}; \vec{R}). \tag{30}$$

The diabatic state is a direct product of the ground state wave function of system B, $\psi^B_{\text{ad},0}(\vec{r}; \vec{R})$, and a rotation of the adiabatic states of system A, $\psi^A_{\text{ad},k}(\vec{r}; \vec{R})$. The matrix elements of $\mathbf{H}_d$ are then given by

$$\begin{aligned}
(\mathbf{H}_d)_{nm} &= \langle \psi_{\text{d},n} | H(\vec{r}, \vec{R}) | \psi_{\text{d},m} \rangle \\
&= \sum_{kl} U^*_{nk} U_{ml} \langle \psi^B_{\text{ad},0} \psi^A_{\text{ad},k} | \; H_A(\vec{r}, \vec{R}) + H_B(\vec{r}, \vec{R}) \; | \psi^B_{\text{ad},0} \psi^A_{\text{ad},l} \rangle \\
&= \sum_{kl} U^*_{nk} U_{ml} \big(E_{k,A} + E_B \big) \delta_{kl} \\
&= \sum_k U^*_{in} U_{mk} \, E_{k,A} + E_B \big(\mathbf{U} \mathbf{U}^\dagger \big)_{mn} \\
&= \mathbf{H}^A_{\text{d},nm} + E_B \, \delta_{nm}.
\end{aligned} \tag{31}$$

Hence the diagonal elements of $\mathbf{H}_d$ each gain the adiabatic energy of B, while the off-diagonal elements remain the same. To understand this result physically, consider that adding a scalar multiplied by the identity yields eigenvalues that are each shifted by the scalar. This means that the eigenvalues of $\mathbf{H}_d$ are each shifted by E_B. Therefore the excitation energy is unchanged, which is the expected behavior upon adding an uncoupled system. Note also that extensivity is sometimes described as doubling the energy when the system size is doubled. However, this definition is too narrow, as it applies to only one adiabatic energy at a time. For example, if subsystem B is a copy of subsystem A, then $E_B = E_{0,A}$, meaning that $E_0 = E_{0,A} + E_B = 2E_{0,A}$, and so the ground state energy is indeed doubled. However, $E_1 = E_{1,A} + E_B = 2E_{1,A} - \text{gap}$, where $\text{gap} = E_{1,A} - E_{0,A}$. Therefore, the excited state energy is not doubled. A more general definition is that the energy of the second subsystem is added to each adiabatic energy.

This analysis shows that the off-diagonal elements of $\mathbf{H}_d$ are intensive. The adiabatic energy gap is also intensive. Intensive here means that adding a subsystem that does not participate in the excitation does not modify the quantity. Such properties can naturally be modeled with an attention mechanism [82–85] that learns the importance of each atom to the excitation. For example, the excitation energy can be modeled as an attention-weighted sum over atomwise quantities. However, as discussed in Supplementary Note 8, this approach led to worse performance than simply predicting extensive energies for each state.

Supplementary Note 16. Proof of diabaticity

Here we prove Eq. (1) in the main text. Using bra-ket notation for the wave functions, we define the diabatic states as a linear combination of adiabatic states,

$$|\psi_{\text{d},n}\rangle = \sum_m |\psi_{\text{ad},m}\rangle \, V^*_{nm}, \tag{32}$$

where $\mathbf{V}$ is a unitary matrix. Multiplying each side by $V_{nn'}$ and summing over n yields

$$|\psi_{\mathrm{ad},n}\rangle = \sum_m |\psi_{\mathrm{d},m}\rangle V_{mn}, \tag{33}$$

where we have renamed the dummy indices, $n \to m$ and $n' \to n$. We have also used the fact that $(\mathbf{V}^\dagger\mathbf{V})_{nm} = \delta_{nm}$ for any unitary matrix, where δ_{nm} is the Kronecker delta. Given the connection between diabatic and adiabatic states, we can relate $\mathbf{V}$ to the derivative coupling $\vec{k}$:

$$\begin{aligned}\vec{k}_{nm} &\equiv \langle\psi_{\mathrm{ad},n}| \nabla_R |\psi_{\mathrm{ad},m}\rangle \\ &= \sum_{ij} V^*_{jn} \left(\nabla_R V_{im} \langle\psi_{\mathrm{d},j}|\psi_{\mathrm{d},i}\rangle + V_{im} \langle\psi_{\mathrm{d},j}| \nabla_R |\psi_{\mathrm{d},i}\rangle\right) \\ &= \sum_i V^*_{in} \nabla_R V_{im}.\end{aligned} \tag{34}$$

We have used the fact that, by definition, the derivative coupling between any two diabatic states is zero. We have also used the orthonormality of the diabatic states:

$$\langle\psi_{\mathrm{d},j}|\psi_{\mathrm{d},i}\rangle = \sum_{nm} V_{jn}V^*_{im} \langle\psi_{\mathrm{ad},n}|\psi_{\mathrm{ad},m}\rangle = \sum_{nm} V_{jn}V^*_{im}\delta_{nm} = \left(\mathbf{V}\mathbf{V}^\dagger\right)_{ij} = \delta_{ij}, \tag{35}$$

which follows from the orthonormality of the adiabatic wave functions. Meanwhile, by construction, the Hamiltonian produced by the model has eigenvalues equal to the adiabatic energies:

$$\begin{aligned}(\mathbf{H}_\mathrm{d})_{nm} &= \left(\mathbf{U}\,\mathrm{diag}(\{E\})\,\mathbf{U}^\dagger\right)_{nm} \\ &= \sum_{ij} U_{ni}\,(E_i\delta_{ij})\,U^*_{mj} \\ &= \left(\sum_i U_{ni}\,\langle\psi_{\mathrm{ad},i}|\right) \hat{H} \left(\sum_j U^*_{mj}\,|\psi_{\mathrm{ad},j}\rangle\right),\end{aligned} \tag{36}$$

where $\mathbf{U}$ is the unitary matrix that diagonalizes $\mathbf{H}_d$, $\hat{H}$ is the Hamiltonian operator, and we have used the relation $\langle\psi_{\mathrm{ad},j}| \hat{H} |\psi_{\mathrm{ad},i}\rangle = E_i \langle\psi_{\mathrm{ad},j}|\psi_{\mathrm{ad},i}\rangle = E_i\delta_{ij}$. Comparing Supplementary Eqs. (36) and (32), we see that $\mathbf{H}_d$ is the representation of $\hat{H}$ in a different electronic basis. In particular, if we choose $\mathbf{U} = \mathbf{V}$, such that $\mathbf{U}$ satisfies Supplementary Eq. (34), then $\mathbf{H}_d$ is the representation of $\hat{H}$ in the diabatic basis.

The model could be trained directly with Supplementary Eq. (34). However, the equation is numerically ill-posed because $\vec{k}_{nm}$ diverges at conical intersections. It is preferable to work instead with the force coupling, $\vec{h}_{nm}$. We now show that Eq. (1) in the main text, which is defined in terms of $\vec{h}_{nm}$, holds only if Supplementary Eq. (34) is satisfied. The left-hand side of Eq. (1) in the main text can be written as

$$\begin{aligned}&\sum_{ij} U^*_{in} \nabla_R \left(\mathbf{U}\,\mathrm{diag}(\{E\})\,\mathbf{U}^\dagger\right)_{ij} U_{jm} \\ &= \sum_{ijk} U^*_{in} \left[(\nabla_R E_k)\,U_{ik}U^*_{jk} + E_k\,(\nabla_R U_{ik})U^*_{jk} + E_k\,U_{ik}(\nabla_R U^*_{jk})\right] U_{jm}.\end{aligned} \tag{37}$$

The first term is

$$\sum_k (\mathbf{U}^\dagger\mathbf{U})_{nk}\,(\nabla_R E_k)\,(\mathbf{U}^\dagger\mathbf{U})_{km} = (\nabla_R E_n)\delta_{nm}, \tag{38}$$

where we have used the fact that $(\mathbf{U}^\dagger\mathbf{U})_{nm} = \delta_{nm}$. Substituting Supplementary Eq. (34) with $\mathbf{V} = \mathbf{U}$, the second term is

$$\sum_k \vec{k}_{nk}(\mathbf{U}^\dagger\mathbf{U})_{km}E_k = \vec{k}_{nm}E_m. \tag{39}$$

Performing the same substitution for the third term gives

$$\sum_k \vec{k}^*_{mk}(\mathbf{U}^\dagger\mathbf{U})_{nk}E_k = -\vec{k}_{nm}E_n, \tag{40}$$

where we have used the anti-Hermitian property $\vec{k}^*_{mn} = -\vec{k}_{nm}$. Adding the three terms gives

$$\left(\mathbf{U}^\dagger(\nabla_R H_d)\mathbf{U}\right)_{nm} = \begin{cases} \nabla_R E_n, & \text{if } n = m, \\ (E_m - E_n)\,\vec{k}_{nm}, & \text{if } n \neq m. \end{cases} \tag{41}$$

Noting that $\vec{f}_n = -\nabla_R E_n$ and $\vec{h}_{nm} = (E_m - E_n)\,\vec{k}_{nm}$ gives Eq. (1) in the main text. Hence Eq. (1) in the main text can only hold if Supplementary Eq. (34) is true. Therefore, enforcing Eq. (1) in the main text ensures that there is no derivative coupling between any pair of diabatic states. Note that in the main text we used three diabatic states, and trained only on energies and couplings between the first two adiabatic states. This still means that all three diabatic states are properly diabatic: if Eq. (1) in the main text is satisfied for even one pair of adiabatic states, then the derivative coupling must be zero between all pairs of diabatic states.

Supplementary Note 17. Training species

Here we provide the motifs and substituents used for combinatorial molecule generation. The literature species used in the test set are given in Supplementary Data 2, together with original publication sources. The literature species used for training, along with their sources, are given in Supplementary Data 3. The sources are given by their number in the references here [32–37, 39, 41, 44, 45, 86–112]. Publication URLs are also provided. Species with a net charge were excluded from training but are provided for completeness. Predicted and experimental quantum yields are given in Supplementary Data 1. For papers that reported the yield at different wavelengths, we chose the wavelength closest to the $n - \pi^*$ (S_1) absorption maximum. Results with both FS surface hopping and the Zhu-Nakamura method are provided.

In some cases only the *cis* or *trans* isomer was actually investigated experimentally, but in all cases we reference the publication for both isomers. Many species in both the training and test set had experimental S_1 yields in non-polar solution. We did not put all such molecules in the test set, because this would mean losing hundreds of thousands of training geometries. Instead we used the 40 species with the fewest QC calculations.

Supplementary Table X. Motifs used for combinatorial species generation. Examples of literature species for each motif are also given. The species numbers are those in Supplementary Data 2 and 3.

Graphs	Literature species
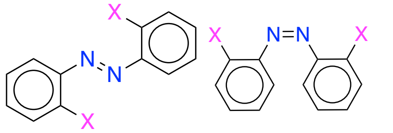	1, 2, 25, 26, 63, 64, 125, 126
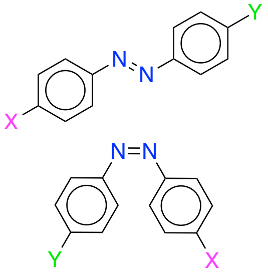	19, 20, 27, 28, 37, 38, 39, 40, 61, 62, 69, 70, 73, 74, 75, 76, 83, 84, 97, 98, 115, 116, 117, 118, 119, 120, 121, 122, 123, 124, 127, 128, 129, 130, 147, 148, 155, 156, 157, 158, 163, 164
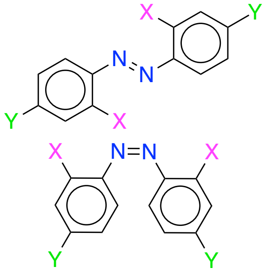	67, 68, 87, 88, 99, 100, 101, 102, 103, 104, 105, 106, 107, 108

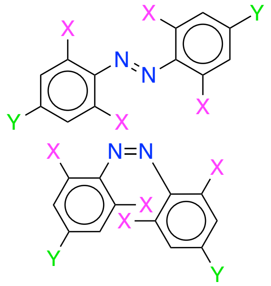	9, 10, 13, 14, 15, 16, 17, 18, 21, 22, 95, 96, 131, 132, 133, 134, 135, 136, 137, 138, 139, 140, 161, 162

Supplementary Table XI. Substituents used for combinatorial species generation. “Azo” denotes the azobenzene attachment site.

SMILES	Graph
[Azo]N(C)Cc1ccccn1	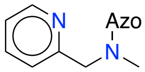
[Azo]N	Azo–NH_2
[Azo]N(CC)Cc1ccccc1	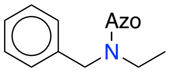
[Azo]C(N)=O	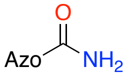
[Azo]OCc1cc(OCc2ccccc2)cc(OCc2ccccc2)c1	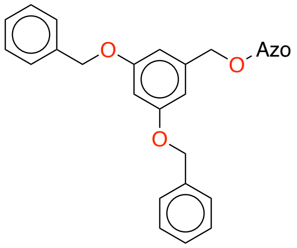
[Azo]N1CCCCC1	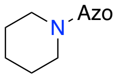
[Azo]c1ccccc1	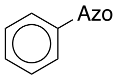
[Azo]NC(=O)CCl	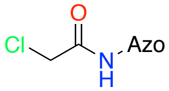
[Azo]OC(C)=O	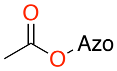
[Azo]N1CCN(C(C)=O)CC1	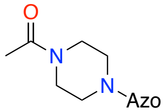
[Azo]NC(=O)C[N+](CC)(CC)CC	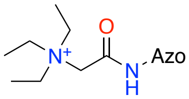
[Azo]Cl	Azo–Cl
[Azo]CO	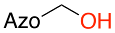
[Azo]C(=O)OCC	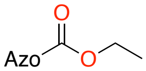
[Azo]CC	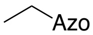
[Azo]C(=O)O	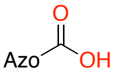
[Azo]C#C	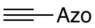

[Azo]NC(=O)CN1C(=O)C=CC1=O	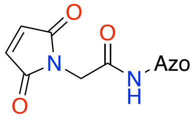
[Azo][N+](=O)[O-]	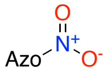
[Azo][N+](C)(C)C	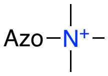
[Azo]O	Azo−OH
[Azo]Oc1c(N)cccc1CC(C)C	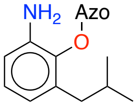
[Azo]F	Azo−F
[Azo]N1CCN(C(=O)CCl)CC1	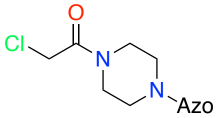
[Azo]Oc1c(N)cccc1C(C)C	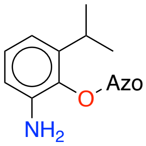
[Azo]NCc1ccccn1	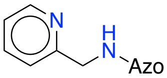
[Azo]OCc1ccccc1	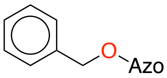
[Azo]NC(C)=O	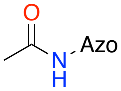
[Azo]c1cn(-c2ccc3cc4ccccc4cc3c2)nn1	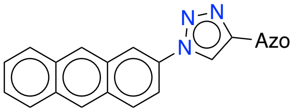
[Azo]C	Azo—
[Azo]C(=O)C(F)(F)F	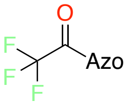
[Azo]N1CCN(C)CC1	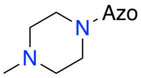
[Azo]c1cn(-c2cccc3ccccc23)nn1	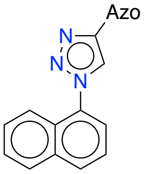

[Azo]c1cn(-c2ccccc2)nn1	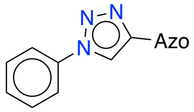
[Azo]C(C)C	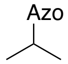
[Azo]C(=O)OC	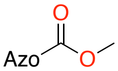
[Azo]N1CCOCC1	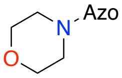
[Azo]N(CC)c1ccccc1	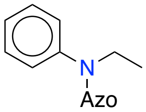
[Azo]N(C)C	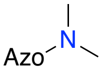
[Azo]N1CCCC1	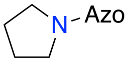
[Azo]N1CC2CN(C(C)=O)CC2C1	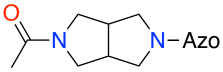
[Azo]C(C)(C)C	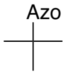
[Azo]NC(=O)C=C	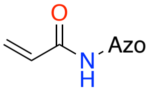
[Azo]N(CCCCCCCC)CCCCCCCC	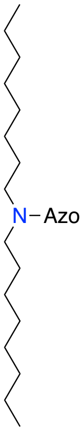
[Azo]OCC	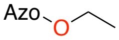
[Azo]N(CC)CC	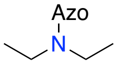

[Azo]N(c1ccc2c(c1)C(C)(C)c1ccccc1-2)c1ccc2c(c1)C(C)(C)c1ccccc1-2	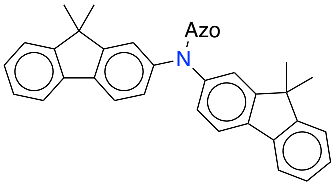
[Azo]NC(=O)CCC[C@H](C[C@H](N)C(=O)O)C(=O)O	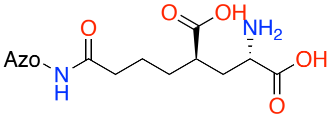

SUPPLEMENTARY REFERENCES

[1] Hanneli R Hudock, Benjamin G Levine, Alexis L Thompson, Helmut Satzger, David Townsend, N Gador, Susanne Ullrich, Albert Stolow, and Todd J Martínez. Ab initio molecular dynamics and time-resolved photoelectron spectroscopy of electronically excited uracil and thymine. *The Journal of Physical Chemistry A*, 111(34):8500–8508, 2007.

[2] John C Tully. Molecular dynamics with electronic transitions. *The Journal of Chemical Physics*, 93(2):1061–1071, 1990.

[3] Sebastian Mai, Philipp Marquetand, and Leticia González. Nonadiabatic dynamics: The SHARC approach. *Wiley Interdisciplinary Reviews: Computational Molecular Science*, 8(6):e1370, 2018.

[4] Michael S Schuurman and David R Yarkony. On the vibronic coupling approximation: A generally applicable approach for determining fully quadratic quasidiabatic coupled electronic state Hamiltonians. *The Journal of chemical physics*, 127 (9):094104, 2007.

[5] John M Herbert, Xing Zhang, Adrian F Morrison, and Jie Liu. Beyond time-dependent density functional theory using only single excitations: Methods for computational studies of excited states in complex systems. *Accounts of chemical research*, 49(5):931–941, 2016.

[6] Julia Westermayr, Michael Gastegger, and Philipp Marquetand. Combining SchNet and SHARC: The SchNarc machine learning approach for excited-state dynamics. *The journal of physical chemistry letters*, 11(10):3828–3834, 2020.

[7] Le Yu, Chao Xu, Yibo Lei, Chaoyuan Zhu, and Zhenyi Wen. Trajectory-based nonadiabatic molecular dynamics without calculating nonadiabatic coupling in the avoided crossing case: Trans$\rightleftharpoons$cis photoisomerization in azobenzene. *Physical Chemistry Chemical Physics*, 16(47):25883–25895, 2014.

[8] TJ Martínez, M Ben-Nun, and RD Levine. Molecular collision dynamics on several electronic states. *The Journal of Physical Chemistry A*, 101(36):6389–6402, 1997.

[9] Eugene S Kryachko and David R Yarkony. Diabatic bases and molecular properties. *International Journal of Quantum Chemistry*, 76(2):235–243, 2000.

[10] Chad E Hoyer, Kelsey Parker, Laura Gagliardi, and Donald G Truhlar. The DQ and DQΦ electronic structure diabatization methods: Validation for general applications. *The Journal of chemical physics*, 144(19):194101, 2016.

[11] Hisao Nakamura and Donald G Truhlar. The direct calculation of diabatic states based on configurational uniformity. *The Journal of Chemical Physics*, 115(22):10353–10372, 2001.

[12] Felix Plasser, Sandra Gómez, Maximilian FSJ Menger, Sebastian Mai, and Leticia González. Highly efficient surface hopping dynamics using a linear vibronic coupling model. *Physical Chemistry Chemical Physics*, 21(1):57–69, 2019.

[13] David MG Williams and Wolfgang Eisfeld. Neural network diabatization: A new ansatz for accurate high-dimensional coupled potential energy surfaces. *The Journal of chemical physics*, 149(20):204106, 2018.

[14] Yafu Guan, Bina Fu, and Dong H Zhang. Construction of diabatic energy surfaces for LiFH with artificial neural networks. *The Journal of chemical physics*, 147(22):224307, 2017.

[15] Yinan Shu and Donald G Truhlar. Diabatization by machine intelligence. *Journal of Chemical Theory and Computation*, 16(10):6456–6464, 2020.

[16] Yinan Shu, Zoltan Varga, Antonio Gustavo Sampaio de Oliveira-Filho, and Donald G Truhlar. Permutationally restrained diabatization by machine intelligence. *Journal of Chemical Theory and Computation*, 17(2):1106–1116, 2021.

[17] Horst Köppel, Joachim Gronki, and Susanta Mahapatra. Construction scheme for regularized diabatic states. *The Journal of Chemical Physics*, 115(6):2377, 2001.

[18] Yafu Guan, Dong H Zhang, Hua Guo, and David R Yarkony. Representation of coupled adiabatic potential energy surfaces using neural network based quasi-diabatic Hamiltonians: 1,2 $^2A'$ states of LiFH. *Physical Chemistry Chemical Physics*, 21 (26):14205–14213, 2019.

[19] Martin Richter, Philipp Marquetand, Jesús González-Vázquez, Ignacio Sola, and Leticia González. SHARC: *ab initio* molecular dynamics with surface hopping in the adiabatic representation including arbitrary couplings. *Journal of chemical theory and computation*, 7(5):1253–1258, 2011.

[20] Erich Runge and Eberhard KU Gross. Density-functional theory for time-dependent systems. *Physical Review Letters*, 52 (12):997, 1984.

[21] Benjamin G Levine, Chaehyuk Ko, Jason Quenneville, and Todd J MartÍnez. Conical intersections and double excitations in time-dependent density functional theory. *Molecular Physics*, 104(5-7):1039–1051, 2006.

[22] Samer Gozem, Federico Melaccio, Alessio Valentini, Michael Filatov, Miquel Huix-Rotllant, Nicolas Ferré, Luis Manuel Frutos, Celestino Angeli, Anna I Krylov, Alexander A Granovsky, et al. Shape of multireference, equation-of-motion coupled-cluster, and density functional theory potential energy surfaces at a conical intersection. *Journal of chemical theory and computation*, 10(8):3074–3084, 2014.

[23] Yihan Shao, Martin Head-Gordon, and Anna I Krylov. The spin–flip approach within time-dependent density functional theory: Theory and applications to diradicals. *The Journal of chemical physics*, 118(11):4807–4818, 2003.

[24] Axel D Becke. A new mixing of Hartree–Fock and local density-functional theories. *The Journal of chemical physics*, 98

(2):1372–1377, 1993.

[25] Michelle M Francl, William J Pietro, Warren J Hehre, J Stephen Binkley, Mark S Gordon, Douglas J DeFrees, and John A Pople. Self-consistent molecular orbital methods. XXIII. A polarization-type basis set for second-row elements. *The Journal of Chemical Physics*, 77(7):3654–3665, 1982.

[26] Michael Filatov. Assessment of density functional methods for obtaining geometries at conical intersections in organic molecules. *Journal of chemical theory and computation*, 9(10):4526–4541, 2013.

[27] Alexander Nikiforov, Jose A Gamez, Walter Thiel, Miquel Huix-Rotllant, and Michael Filatov. Assessment of approximate computational methods for conical intersections and branching plane vectors in organic molecules. *The Journal of chemical physics*, 141(12):124122, 2014.

[28] Ling Yue, Yajun Liu, and Chaoyuan Zhu. Performance of TDDFT with and without spin-flip in trajectory surface hopping dynamics: cis$\rightleftharpoons$trans azobenzene photoisomerization. *Physical Chemistry Chemical Physics*, 20(37):24123–24139, 2018.

[29] Yihan Shao, Zhengting Gan, Evgeny Epifanovsky, Andrew TB Gilbert, Michael Wormit, Joerg Kussmann, Adrian W Lange, Andrew Behn, Jia Deng, Xintian Feng, et al. Advances in molecular quantum chemistry contained in the Q-Chem 4 program package. *Molecular Physics*, 113(2):184–215, 2015.

[30] HM Dhammika Bandara and Shawn C Burdette. Photoisomerization in different classes of azobenzene. *Chemical Society Reviews*, 41(5):1809–1825, 2012.

[31] Jingbai Li, Patrick Reiser, Benjamin R Boswell, André Eberhard, Noah Z Burns, Pascal Friederich, and Steven A Lopez. Automatic discovery of photoisomerization mechanisms with nanosecond machine learning photodynamics simulations. *Chemical science*, 12(14):5302–5314, 2021.

[32] Shmuel Malkin and Ernst Fischer. Temperature dependence of photoisomerization. Part II. Quantum yields of cis-trans isomerizations in azo-compounds. *The Journal of Physical Chemistry*, 66(12):2482–2486, 1962.

[33] Hermann Rau and Shen Yu-Quan. Photoisomerization of sterically hindered azobenzenes. *Journal of Photochemistry and Photobiology A: Chemistry*, 42(2-3):321–327, 1988.

[34] Gerhard J Mohr and Ulrich-W Grummt. Photochemistry of the amine-sensor dye 4-N, N-dioctylamino-4'-trifluoroacetylazobenzene. *Journal of Photochemistry and Photobiology A: Chemistry*, 163(3):341–345, 2004.

[35] Christopher Knie, Manuel Utecht, Fangli Zhao, Hannes Kulla, Sergey Kovalenko, Albert M Brouwer, Peter Saalfrank, Stefan Hecht, and David Bléger. *ortho*-Fluoroazobenzenes: Visible light switches with very long-lived Z isomers. *Chemistry–A European Journal*, 20(50):16492–16501, 2014.

[36] J Moreno, M Gerecke, AL Dobryakov, IN Ioffe, AA Granovsky, D Bléger, S Hecht, and SA Kovalenko. Two-photon-induced versus one-photon-induced isomerization dynamics of a bistable azobenzene derivative in solution. *The Journal of Physical Chemistry B*, 119(37):12281–12288, 2015.

[37] Hermann Rau. Further evidence for rotation in the π, π^* and inversion in the n, π^* photoisomerization of azobenzenes. *Journal of photochemistry*, 26(2-3):221–225, 1984.

[38] Ernst Fischer. Calculation of photostationary states in systems A $\rightleftharpoons$ B when only A is known. *The Journal of Physical Chemistry*, 71(11):3704–3706, 1967.

[39] George Zimmerman, Lue-Yung Chow, and Un-Jin Paik. The photochemical isomerization of azobenzene. *Journal of the American Chemical Society*, 80(14):3528–3531, 1958.

[40] Philipp Marquetand, Martin Richter, Jesús González-Vázquez, Ignacio Sola, and Leticia González. Nonadiabatic ab initio molecular dynamics including spin–orbit coupling and laser fields. *Faraday discussions*, 153:261–273, 2011.

[41] Pietro Bortolus and Sandra Monti. Cis-trans photoisomerization of azobenzene. Solvent and triplet donors effects. *Journal of Physical Chemistry*, 83(6):648–652, 1979.

[42] Jacques Ronayette, René Arnaud, Philippe Lebourgeois, and Jacques Lemaire. Isomérisation photochimique de l'azobenzène en solution. I. *Canadian Journal of Chemistry*, 52(10):1848–1857, 1974.

[43] Pietro Bortolus and Sandra Monti. Cis $\rightleftharpoons$ trans photoisomerization of azobenzene-cyclodextrin inclusion complexes. *Journal of Physical Chemistry*, 91(19):5046–5050, 1987.

[44] HM Dhammika Bandara, Tracey R Friss, Miriam M Enriquez, William Isley, Christopher Incarvito, Harry A Frank, Jose Gascon, and Shawn C Burdette. Proof for the concerted inversion mechanism in the trans$\rightarrow$cis isomerization of azobenzene using hydrogen bonding to induce isomer locking. *The Journal of organic chemistry*, 75(14):4817–4827, 2010.

[45] HM Dhammika Bandara, Shannon Cawley, José A Gascón, and Shawn C Burdette. Short-circuiting azobenzene photoisomerization with electron-donating substituents and reactivating the photochemistry with chemical modification, 2011.

[46] Giustiniano Tiberio, Luca Muccioli, Roberto Berardi, and Claudio Zannoni. How does the trans-cis photoisomerization of azobenzene take place in organic solvents? *ChemPhysChem*, 11(5):1018, 2010.

[47] Giovanni Granucci and Maurizio Persico. Excited state dynamics with the direct trajectory surface hopping method: azobenzene and its derivatives as a case study. *Theoretical Chemistry Accounts*, 117(5):1131–1143, 2007.

[48] Jimmy K Yu, Christoph Bannwarth, Ruibin Liang, Edward G Hohenstein, and Todd J Martínez. Nonadiabatic dynamics simulation of the wavelength-dependent photochemistry of azobenzene excited to the nπ^* and $\pi\pi^*$ excited states. *Journal of the American Chemical Society*, 142(49):20680–20690, 2020.

[49] Brian R Landry and Joseph E Subotnik. How to recover Marcus theory with fewest switches surface hopping: Add just a touch of decoherence. *The Journal of chemical physics*, 137(22):22A513, 2012.

[50] Hsing-Ta Chen and David R Reichman. On the accuracy of surface hopping dynamics in condensed phase non-adiabatic problems. *The Journal of Chemical Physics*, 144(9):094104, 2016.

[51] J Patrick Zobel, Moritz Heindl, Felix Plasser, Sebastian Mai, and Leticia González. Surface hopping dynamics on vibronic coupling models. *Accounts of chemical research*, 54(20):3760–3771, 2021.

[52] Lea M Ibele, Yorick Lassmann, Todd J Martínez, and Basile FE Curchod. Comparing (stochastic-selection) ab initio multiple spawning with trajectory surface hopping for the photodynamics of cyclopropanone, fulvene, and dithiane. *The Journal of Chemical Physics*, 154(10):104110, 2021.

[53] Adam Paszke, Sam Gross, Francisco Massa, Adam Lerer, James Bradbury, Gregory Chanan, Trevor Killeen, Zeming Lin, Natalia Gimelshein, Luca Antiga, Alban Desmaison, Andreas Kopf, Edward Yang, Zachary DeVito, Martin Raison, Alykhan Tejani, Sasank Chilamkurthy, Benoit Steiner, Lu Fang, Junjie Bai, and Soumith Chintala. PyTorch: An imperative style, high-performance deep learning library. In *Advances in Neural Information Processing Systems 32*, pages 8024–8035. 2019.

[54] Kristof Schütt, Oliver Unke, and Michael Gastegger. Equivariant message passing for the prediction of tensorial properties and molecular spectra. In *International Conference on Machine Learning*, pages 9377–9388. PMLR, 2021.

[55] Johannes Gasteiger, Janek Groß, and Stephan Günnemann. Directional message passing for molecular graphs. In *International Conference on Learning Representations*, 2020. URL `https://openreview.net/forum?id=B1eWbxStPH`.

[56] Wen-Kai Chen, Xiang-Yang Liu, Wei-Hai Fang, Pavlo O Dral, and Ganglong Cui. Deep learning for nonadiabatic excited-state dynamics. *The journal of physical chemistry letters*, 9(23):6702–6708, 2018.

[57] Pavlo O Dral, Mario Barbatti, and Walter Thiel. Nonadiabatic excited-state dynamics with machine learning. *The journal of physical chemistry letters*, 9(19):5660–5663, 2018.

[58] Deping Hu, Yu Xie, Xusong Li, Lingyue Li, and Zhenggang Lan. Inclusion of machine learning kernel ridge regression potential energy surfaces in on-the-fly nonadiabatic molecular dynamics simulation. *The journal of physical chemistry letters*, 9(11):2725–2732, 2018.

[59] Julia Westermayr, Michael Gastegger, Maximilian FSJ Menger, Sebastian Mai, Leticia González, and Philipp Marquetand. Machine learning enables long time scale molecular photodynamics simulations. *Chemical science*, 10(35):8100–8107, 2019.

[60] Julia Westermayr and Philipp Marquetand. Machine learning for electronically excited states of molecules. *Chemical Reviews*, 121(16):9873–9926, 2020.

[61] Julia Westermayr and Philipp Marquetand. Deep learning for UV absorption spectra with SchNarc: First steps toward transferability in chemical compound space. *The Journal of Chemical Physics*, 153(15):154112, 2020.

[62] Daniel Schwalbe-Koda, Aik Rui Tan, and Rafael Gómez-Bombarelli. Differentiable sampling of molecular geometries with uncertainty-based adversarial attacks. *Nature communications*, 12(1):1–12, 2021.

[63] Mario Barbatti and Kakali Sen. Effects of different initial condition samplings on photodynamics and spectrum of pyrrole. *International Journal of Quantum Chemistry*, 116(10):762–771, 2016.

[64] Shuichi Nosé. A unified formulation of the constant temperature molecular dynamics methods. *The Journal of chemical physics*, 81(1):511–519, 1984.

[65] William G Hoover. Canonical dynamics: Equilibrium phase-space distributions. *Physical review A*, 31(3):1695, 1985.

[66] Ask Hjorth Larsen, Jens Jørgen Mortensen, Jakob Blomqvist, Ivano E Castelli, Rune Christensen, Marcin Dułak, Jesper Friis, Michael N Groves, Bjørk Hammer, Cory Hargus, Eric D Hermes, Paul C Jennings, Peter Bjerre Jensen, James Kermode, John R Kitchin, Esben Leonhard Kolsbjerg, Joseph Kubal, Kristen Kaasbjerg, Steen Lysgaard, Jón Bergmann Maronsson, Tristan Maxson, Thomas Olsen, Lars Pastewka, Andrew Peterson, Carsten Rostgaard, Jakob Schiøtz, Ole Schütt, Mikkel Strange, Kristian S Thygesen, Tejs Vegge, Lasse Vilhelmsen, Michael Walter, Zhenhua Zeng, and Karsten W Jacobsen. The atomic simulation environment—a Python library for working with atoms. *Journal of Physics: Condensed Matter*, 29(27):273002, 2017.

[67] Loup Verlet. Computer "experiments" on classical fluids. I. Thermodynamical properties of Lennard-Jones molecules. *Physical review*, 159(1):98, 1967.

[68] Ling Yue, Le Yu, Chao Xu, Yibo Lei, Yajun Liu, and Chaoyuan Zhu. Benchmark performance of global switching versus local switching for trajectory surface hopping molecular dynamics simulation: Cis⇌trans azobenzene photoisomerization. *ChemPhysChem*, 18(10):1274–1287, 2017.

[69] Sebastian Mai, Philipp Marquetand, and Leticia González. A general method to describe intersystem crossing dynamics in trajectory surface hopping. *International Journal of Quantum Chemistry*, 115(18):1215–1231, 2015.

[70] Felix Plasser, Giovanni Granucci, Jiri Pittner, Mario Barbatti, Maurizio Persico, and Hans Lischka. Surface hopping dynamics using a locally diabatic formalism: Charge transfer in the ethylene dimer cation and excited state dynamics in the 2-pyridone dimer. *The Journal of chemical physics*, 137(22):22A514, 2012.

[71] Neural Force Field. `https://github.com/learningmatter-mit/NeuralForceField`.

[72] Giovanni Granucci and Maurizio Persico. Critical appraisal of the fewest switches algorithm for surface hopping. *The Journal of chemical physics*, 126(13):134114, 2007.

[73] Wujie Wang, Tzuhsiung Yang, William H. Harris, and Rafael Gómez-Bombarelli. Active learning and neural network potentials accelerate molecular screening of ether-based solvate ionic liquids. *Chemical Communications*, 56(63):8920, 2020.

[74] Shi Jun Ang, Wujie Wang, Daniel Schwalbe-Koda, Simon Axelrod, and Rafael Gómez-Bombarelli. Active learning accelerates ab initio molecular dynamics on reactive energy surfaces. *Chem*, 7(3):738, 2021.

[75] Axel D Becke. Density-functional exchange-energy approximation with correct asymptotic behavior. *Physical review A*, 38 (6):3098, 1988.

[76] John P Perdew. Density-functional approximation for the correlation energy of the inhomogeneous electron gas. *Physical Review B*, 33(12):8822, 1986.

[77] Troy Van Voorhis and Martin Head-Gordon. A geometric approach to direct minimization. *Molecular Physics*, 100(11): 1713–1721, 2002.

[78] Justin S Smith, Benjamin T Nebgen, Roman Zubatyuk, Nicholas Lubbers, Christian Devereux, Kipton Barros, Sergei Tretiak, Olexandr Isayev, and Adrian E Roitberg. Approaching coupled cluster accuracy with a general-purpose neural network potential through transfer learning. *Nature communications*, 10(1):1–8, 2019.

[79] Michael Filatov. Spin-restricted ensemble-referenced Kohn–Sham method: basic principles and application to strongly correlated ground and excited states of molecules. *Wiley Interdisciplinary Reviews: Computational Molecular Science*, 5 (1):146–167, 2015.

[80] Seunghoon Lee, Michael Filatov, Sangyoub Lee, and Cheol Ho Choi. Eliminating spin-contamination of spin-flip time dependent density functional theory within linear response formalism by the use of zeroth-order mixed-reference (MR) reduced density matrix. *The Journal of chemical physics*, 149(10):104101, 2018.

[81] Jimmy K Yu, Christoph Bannwarth, Edward G Hohenstein, and Todd J Martínez. *Ab initio* nonadiabatic molecular dynamics with hole–hole Tamm–Dancoff approximated density functional theory. *Journal of Chemical Theory and Computation*, 16(9):5499–5511, 2020.

[82] Dzmitry Bahdanau, Kyung Hyun Cho, and Yoshua Bengio. Neural machine translation by jointly learning to align and translate. In *3rd International Conference on Learning Representations, ICLR 2015*, 2015.

[83] Yoon Kim, Carl Denton, Luong Hoang, and Alexander M Rush. Structured attention networks. *arXiv preprint arXiv:1702.00887*, 2017.

[84] Ashish Vaswani, Noam Shazeer, Niki Parmar, Jakob Uszkoreit, Llion Jones, Aidan N Gomez, Łukasz Kaiser, and Illia Polosukhin. Attention is all you need. In *Advances in neural information processing systems*, pages 5998–6008, 2017.

[85] Petar Veličković, Guillem Cucurull, Arantxa Casanova, Adriana Romero, Pietro Liò, and Yoshua Bengio. Graph attention networks. In *International Conference on Learning Representations*, 2018. URL `https://openreview.net/forum?id=rJXMpikCZ`.

[86] Motoki Kurita, Miku Makihara, and Hideyuki Nakano. Photochromic reactions of 4-[bis (9, 9-dimethylfluoren-2-yl) amino] azobenzene in low molecular-mass organogels. *Soft materials*, 12(1):42–46, 2014.

[87] Dina Gegiou, KA Muszkat, and Ernst Fischer. Temperature dependence of photoisomerization. V. Effect of substituents on the photoisomerization of stilbenes and azobenzenes. *Journal of the American Chemical Society*, 90(15):3907–3918, 1968.

[88] Paul Sierocki, Huub Maas, Patrick Dragut, Gabriele Richardt, Fritz Vögtle, Luisa De Cola, Fred Brouwer, and Jeffrey I Zink. Photoisomerization of azobenzene derivatives in nanostructured silica. *The Journal of Physical Chemistry B*, 110 (48):24390–24398, 2006.

[89] Ron Siewertsen, Hendrikje Neumann, Bengt Buchheim-Stehn, Rainer Herges, Christian Nather, Falk Renth, and Friedrich Temps. Highly efficient reversible Z-E photoisomerization of a bridged azobenzene with visible light through resolved $S_1(n\pi^*)$ absorption bands. *Journal of the American Chemical Society*, 131(43):15594–15595, 2009.

[90] Pascal Lentes, Eduard Stadler, Fynn Röhricht, Arne Brahms, Jens Gröbner, Frank D Sönnichsen, Georg Gescheidt, and Rainer Herges. Nitrogen bridged diazocines: Photochromes switching within the near-infrared region with high quantum yields in organic solvents and in water. *Journal of the American Chemical Society*, 141(34):13592–13600, 2019.

[91] PP Birnbaum and DWG Style. The photo-isomerization of some azobenzene derivatives. *Transactions of the Faraday Society*, 50:1192–1196, 1954.

[92] S Anitha Nagamani, Yasuo Norikane, and Nobuyuki Tamaoki. Photoinduced hinge-like molecular motion: studies on xanthene-based cyclic azobenzene dimers. *The Journal of organic chemistry*, 70(23):9304–9313, 2005.

[93] John Olmsted III, Jerry Lawrence, and Geary G Yee. Photochemical storage potential of azobenzenes. *Solar Energy*, 30 (3):271–274, 1983.

[94] Tao Chen, Atsushi Yamaguchi, Kazumasa Igarashi, Naoya Nakagawa, Hidenori Nishioka, Hiroyuki Asanuma, and Mikio Yamashita. Ultrafast photoisomerization and its single-shot pump pulse efficiency of trans-azobenzene derivative: Compound for photosensitive DNA. *Optics Communications*, 285(6):1206–1211, 2012.

[95] Alexis Goulet-Hanssens, T Christopher Corkery, Arri Priimagi, and Christopher J Barrett. Effect of head group size on the photoswitching applications of azobenzene Disperse Red 1 analogues. *Journal of Materials Chemistry C*, 2(36):7505–7512, 2014.

[96] Alexandre Mourot, Michael A Kienzler, Matthew R Banghart, Timm Fehrentz, Florian ME Huber, Marco Stein, Richard H Kramer, and Dirk Trauner. Tuning photochromic ion channel blockers. *ACS chemical neuroscience*, 2(9):536–543, 2011.

[97] Oleg Sadovski, Andrew A Beharry, Fuzhong Zhang, and G Andrew Woolley. Spectral tuning of azobenzene photoswitches for biological applications. *Angewandte Chemie International Edition*, 48(8):1484–1486, 2009.

[98] Subhas Samanta, Chuanguang Qin, Alan J Lough, and G Andrew Woolley. Bidirectional photocontrol of peptide conformation with a bridged azobenzene derivative. *Angewandte Chemie International Edition*, 51(26):6452–6455, 2012.

[99] Subhas Samanta, Andrew A Beharry, Oleg Sadovski, Theresa M McCormick, Amirhossein Babalhavaeji, Vince Tropepe, and G Andrew Woolley. Photoswitching azo compounds in vivo with red light. *Journal of the American Chemical Society*, 135(26):9777–9784, 2013.

[100] Marta Gascón-Moya, Arnau Pejoan, Mercè Izquierdo-Serra, Silvia Pittolo, Gisela Cabré, Jordi Hernando, Ramon Alibés, Pau Gorostiza, and Félix Busqué. An optimized glutamate receptor photoswitch with sensitized azobenzene isomerization. *The Journal of organic chemistry*, 80(20):9915–9925, 2015.

[101] Hermann Rau, Gerhard Greiner, Guenter Gauglitz, and Herbert Meier. Photochemical quantum yields in the A $(+h\nu)$ $\rightleftharpoons$ B $(+h\nu, \Delta)$ system when only the spectrum of A is known. *Journal of physical chemistry*, 94(17):6523–6524, 1990.

[102] Angelo Albini, Elisa Fasani, and Silvio Pietra. The photochemistry of azo dyes. Photoisomerisation versus photoreduction from 4-diethylaminoazobenzene and 4-diethylamino-4'-methoxyazobenzene. *Journal of the Chemical Society, Perkin Transactions 2*, (7):1021–1024, 1983.

[103] Yu Jin Lee, Sung Ik Yang, Dae Seung Kang, and Sang-Woo Joo. Solvent dependent photo-isomerization of 4-dimethylaminoazobenzene carboxylic acid. *Chemical Physics*, 361(3):176–179, 2009.

[104] FO Koller, C Sobotta, TE Schrader, T Cordes, WJ Schreier, A Sieg, and P Gilch. Slower processes of the ultrafast photo-isomerization of an azobenzene observed by IR spectroscopy. *Chemical Physics*, 341(1-3):258–266, 2007.

[105] Tetsuro Umemoto, Yuta Ohtani, Takamasa Tsukamoto, Tetsuya Shimada, and Shinsuke Takagi. Pinning effect for photoisomerization of a dicationic azobenzene derivative by anionic sites of the clay surface. *Chemical Communications*, 50 (3):314–316, 2014.

[106] Ruriko Tahara, Tatsuya Morozumi, Hiroshi Nakamura, and Masatsugu Shimomura. Photoisomerization of azobenzocrown ethers. Effect of complexation of alkaline earth metal ions. *The Journal of Physical Chemistry B*, 101(39):7736–7743, 1997.

[107] M Dong, A Babalhavaeji, MJ Hansen, L Kalman, and GA Woolley. Red, far-red, and near infrared photoswitches based on azonium ions. *Chemical Communications*, 51(65):12981–12984, 2015.

[108] Subhas Samanta, Amirhossein Babalhavaeji, Ming-xin Dong, and G Andrew Woolley. Photoswitching of ortho-substituted azonium ions by red light in whole blood. *Angewandte Chemie*, 125(52):14377–14380, 2013.

[109] Elizabeth C Carroll, Shai Berlin, Joshua Levitz, Michael A Kienzler, Zhe Yuan, Dorte Madsen, Delmar S Larsen, and Ehud Y Isacoff. Two-photon brightness of azobenzene photoswitches designed for glutamate receptor optogenetics. *Proceedings of the National Academy of Sciences*, 112(7):E776–E785, 2015.

[110] Andreas Archut, Fritz Vögtle, Luisa De Cola, Gianluca Camillo Azzellini, Vincenzo Balzani, PS Ramanujam, and Rolf H Berg. Azobenzene-functionalized cascade molecules: photoswitchable supramolecular systems. *Chemistry–A European Journal*, 4(4):699–706, 1998.

[111] Igor K Lednev, Tian-Qing Ye, Laurence C Abbott, Ronald E Hester, and John N Moore. Photoisomerization of a capped azobenzene in solution probed by ultrafast time-resolved electronic absorption spectroscopy. *The Journal of Physical Chemistry A*, 102(46):9161–9166, 1998.

[112] Andrew A Beharry, Oleg Sadovski, and G Andrew Woolley. Photo-control of peptide conformation on a timescale of seconds with a conformationally constrained, blue-absorbing, photo-switchable linker. *Organic & biomolecular chemistry*, 6(23):4323–4332, 2008.
