# Supplementary material for: Excited state non-adiabatic dynamics of large photoswitchable molecules using a chemically transferable machine learning potential
Source: Nat Commun. 2022 Jun 15;13:3440. doi: 10.1038/s41467-022-30999-w (PMC9200747; doi:10.1038/s41467-022-30999-w)
Supplement: Supplementary file 2 — Description of Additional Supplementary Files [file 41467_2022_30999_MOESM2_ESM.docx]

File Name: Supplementary Data 1

Description: Predicted and experimental quantum yields of azobenzene derivatives. Results are provided for *cis* molecules, *trans* molecules, and both. Results from both Tully’s surface hopping and the Zhu-Nakamura method are included.

File Name: Supplementary Data 2

Description: Test set. These literature molecules were held out from training, and their predicted yields were compared with experiment. Molecule numbering, SMILES strings, and original sources are provided.

File Name: Supplementary Data 3

Description: Training set. These literature molecules were used for training. Molecule numbering, SMILES strings, and original sources are provided.
